# Exploring psychometric properties of the Strengths and Difficulties Questionnaire (SDQ) among adolescent Palestinian refugee girls in Palestine and Jordan

**DOI:** 10.1371/journal.pone.0349999

**Published:** 2026-09-25

**Authors:** Mohammed B. A. Sarhan, Rula Ghandour, Weeam Hammoudeh

**Affiliations:** 1 Graduate School of Health Management, Keio University, Fujisawa, Kanagawa, Japan; 2 Institute of Community and Public Health, Birzeit University, Birzeit, Occupied Palestinian territory; 3 Hunter College, The City University of New York, New York, United States of America; Amrita Vishwa Vidyapeetham - Amritapuri Campus, INDIA

## Abstract

**Background:**

The Strengths and Difficulties Questionnaire (SDQ) is a commonly used screening instrument for assessing emotional and behavioral strengths and difficulties among children and adolescents. However, although the SDQ’s psychometric properties have been tested extensively in the literature, its current form is under debate as to whether it is suitable as a tool to assess adolescent mental health, especially among refugees. Therefore, this study aims to validate the SDQ scale among Adolescent Palestinian refugees in the West Bank, Palestine, and Jordan.

**Methods:**

The psychometric properties of the original SDQ and other suggested alternatives were tested. First, construct validity for the original SDQ was tested through confirmatory factor analysis (CFA). Then, exploratory factor analysis (EFA) was used to evaluate the latent factorial structure for suggested alternatives, followed by CFA to provide additional evaluation of each alternative. Then, convergent, discriminant, and criterion validity were assessed. Finally, the internal consistency and reliability were assessed using multiple tests.

**Results:**

This study included 2,828 adolescent girls living in Palestine refugee camps in the West Bank and Jordan. Of these, 2,725 participants were included in the final analysis based on completion of the SDQ. CFA indicated that the original five-factor SDQ demonstrated poor fit in this sample (CFI/TLI < 0.40; RMSEA = 0.081; SRMR = 0.110), with several items showing low standardized loadings. EFA and CFA analyses supported alternative factorial structures. A four-factor model showed acceptable fit (CFI = 0.896; RMSEA = 0.040; SRMR = 0.039) after removing poorly performing items, but a three-factor solution provided the best overall fit (CFI = 0.942; TLI = 0.930; RMSEA = 0.034; SRMR = 0.033). The final three-factor model retained 15 items representing Emotional Symptoms, Prosocial behavior, and Conduct Problems. The weak correlation between the difficulties did not support convergent validity score and WHO-5 well-being (r = −0.245). Criterion validity analyses showed acceptable discrimination for overall difficulties (AUC = 0.776). Reliability analyses indicated adequate internal consistency for the composite difficulties scale (α = 0.71).

**Conclusion:**

The original SDQ structure validity and reliability were not supported for use among Palestinian refugees in the West Bank and Jordan. Instead, the 3-factor and 15-item alternative might be a better alternative, given its superior model fit indices and construct validity. However, it still had limitations in reliability and convergent validity, making it far from being an adequate substitute for the original SDQ. Contexts, culture, and the surrounding environment might influence how adolescents in refugee settings understand, interpret, and respond to SDQ items.

## Introduction

Adolescence is a critical developmental stage for mental well-being because it represents a period of transition. During this period, questions of identity and personhood become more salient, and social and emotional habits are established [1]. The changes that occur during adolescence can pose challenges to adolescents’ mental health [2,3], and may affect their emotions, behaviors, and cognitive abilities [2]. Consequently, concern about child and adolescent mental health has come to the forefront in recent years. According to the World Health Organization (WHO), based on data from the Global Burden of Disease 2021 [1], mental health disorders account for 15% of the global burden of disease among adolescents.

Increasingly, research has highlighted an alarming rise in adolescent and youth mental health problems, particularly over the last decade [4]. Anxiety disorders, depression, conduct disorders, and eating disorders (e.g., anorexia), among others, are becoming increasingly prevalent among this age group. For example, the global incidence of anxiety disorders among individuals aged 10–24 years increased by approximately 52% between 1990 and 2021 [5]. Numerous factors contribute to the poor mental health of adolescents, including poverty, exposure to violence, abuse, stigma, discrimination, and poor home conditions, among others [1].

The rise in mental health concerns and disorders among adolescents is of even greater importance given the long-term impacts of poor mental health during adolescence on health throughout a person’s life [1]. These long-term impacts may be substantial when mental health challenges are not addressed and disorders are left undiagnosed or not treated [1] in a timely and appropriate manner [6]. Early detection and preventive interventions are critical to addressing adolescent mental health needs [7,8]. Therefore, valid and reliable screening instruments are essential for detecting psychological, emotional, and behavioral problems early enough to enable timely intervention and prevent any long-term consequences [9].

The Strengths and Difficulties Questionnaire (SDQ; https://www.sdqinfo.org/) is one of the most widely used standardized screening instruments for assessing emotional and behavioral strengths and difficulties among children and adolescents. It employs a brief, multi-domain questionnaire that captures both positive attributes and problem behaviors [3]. The SDQ is widely used for several reasons. First, it can be used for mental health screening in clinical settings and research [10,11]. Second, it is relatively brief, consisting of only 25 items. Third, it assesses multiple constructs, including difficulties related to conduct problems, emotional symptoms, peer relationship problems, and hyperactivity, as well as strengths primarily associated with prosocial behaviors [11]. Studies have demonstrated that SDQ subscales are associated with conceptually similar Diagnostic and Statistical Manual of Mental Disorders (DSM) diagnoses, suggesting that the instrument may be useful for identifying people at risk for disorders such as depression, anxiety, and attention-deficit/hyperactivity disorder (ADHD), among others [10]. Finally, multiple informant versions are available, enabling the collection of data from parents, teachers, and adolescents themselves [11]. The SDQ has also been translated and validated in several languages.

Despite its widespread use and the extensive evaluation of its psychometric properties, debate remains regarding whether the current form of the SDQ is appropriate for assessing adolescent mental health [3,11,12]. Although the SDQ has been extensively applied in both research and clinical purposes across diverse populations [3], its performance among adolescents has produced inconsistent findings. For example, the SDQ demonstrated promising results in assessing the mental health of newly settled Iraqi refugees, with changes in scores over time, indicating that timing and contextual factors may influence SDQ outcomes [13]. In contrast, a study conducted in Norway reported weak factor loadings and low internal consistency among Afghan, Syrian, Albanian, Eritrean, and Somali refugee adolescents [14]. Consequently, the current self-report version of the SDQ may not be suitable for cross-country comparisons, and further revisions, testing, and validation across different countries, contexts, and populations are needed [12].

Furthermore, concerns have been raised regarding the SDQ’s unstable factor structure, low reliability, and problematic item-wording. Several alternative factor structures have been proposed over the years. Specifically, three-, four-, and six-factor models have been reported to demonstrate a more stable structure than the original five-factor model [3,11,15–18]. In addition, subscales such as hyperactivity-inattention and peer problems have been found to lack uni-dimensionality, indicating poor structural fit [3]. Several studies have also reported that reverse-worded ‘difficulties’ items load on the prosocial behavior factor [11,19]. Nonetheless, specific items, such as “I usually do as I am told” (being obedient) and “I get on better with adults than with people my own age” (adults) have been criticized for their negative impact on model fit and for their limited. Finally, low reliability has been reported across all SDQ dimensions in multiple studies [3,20].

The development of the SDQ and similar instruments stems from a recognition of the importance of having reliable and valid measures to assess mental health. Reliable screening tools are particularly important for identifying people at-risk, especially in settings where adolescents are exposed to prolonged political instability, structural violence, and other factors that increase their vulnerability to mental health challenges. This is particularly relevant to Palestinian refugee adolescents, who are exposed to various mental health stressors [21–23].

Palestinian refugees living in overcrowded refugee camps face various structural and environmental disadvantages, including poverty, limited access to clean water, inadequate social infrastructure, and unhealthy and unsafe living conditions [23,24]. These adverse circumstances and chronic stressors increase their vulnerability to mental health disorders [21,23]. A study of students aged 11–16 years attending the United Nations Relief and Works Agency (UNRWA) schools in Palestine, Syria, Lebanon, and Jordan reported alarmingly high rates of mental health problems. Approximately 57% them reported being lonely, 37% reported excessive worrying, and around 20% reported suicidal ideation [22]. Similarly, another study found that the prevalence of suicidal ideation was 27% among Palestinian middle-school students attending UNRWA schools in Jordan and 27.2% among their counterparts in the West Bank [25]. Poor mental health status has also been shown to affect adolescents’ daily lives. For example, it has been associated with higher rates of school absenteeism [22]. Furthermore, a study conducted in the West Bank found that adolescents aged 12–19 years had experienced high levels of trauma, with prolonged direct and indirect exposure to occupation-related stressors and adverse economic conditions negatively affecting their mental well-being [26].

The SDQ has been proposed as a potentially useful tool for assessing adolescent mental health in the Palestinian context. Thabet et. al [27] were the first to apply the SDQ among Palestinians in Gaza in 2000. They conducted a series of factor analyses to confirm whether the questionnaire exhibited the same factor as that reported in previous validation studies conducted in Western countries. Although the authors concluded that the SDQ showed promise for use in Palestine, they also identified concerns regarding multiple items, such as feeling unhappy, feeling scared, being distracted (distractibility), stealing, and being picked on or bullied. They suggested that these items may carry different meanings in the Palestinian cultural context than in Western communities. Furthermore, they concluded that the emotional and peer relationship problems were heterogeneous and not unidimensional, in contrast to findings reported in Western studies [27].

Since Thabet et. al.’s study in 2000, several studies have reported using the SDQ among Palestinian adolescents [27–31]. During this period, Palestinians have experienced major events such as the Second Intifada or Uprising, multiple wars on Gaza, the COVID-19 pandemic, and other crises that have profoundly affected the Palestinian community, particularly adolescents and young people [32–34]. These experiences may have shaped the perspectives of the current generation and, could consequently impact their underlying interpretations of SDQ items and constructs. This highlights the need to examine the instrument’s validity in this context. There were some attempts in the literature to do so. For example, one study reported eliminating several items, such as obedient, to improve Cronbach’s alpha [29]. However, this study and others did not report conducting any psychometric testing of the SDQ structure [28 31].

According to Veronese et. al., the SDQ has never been formally validated in Palestine [31], especially among refugee populations, despite its use in several studies. This represents an important gap in the literature, given that Palestinian refugees residing in the West Bank, Gaza, and Jordan continue to experience chronic trauma and multiple psychosocial stressors. Additional investigation of the SDQ’s structure, validity, and reliability remains warranted despite its widespread use [35]. Moreover, the number of validation studies conducted in comparable settings remains limited, and no gold-standard screening instrument exists for identifying adolescent mental health problems in these contexts. This study aims to examine whether the original SDQ structure is valid and reliable and therefore suitable to be used among Palestinian refugee adolescents in the West Bank, Palestine, and Jordan. This study also aims, when necessary, to propose a better-fitting alternative SDQ model that is more sensitive to this population’s culture and social values. In summary, the research question that this manuscript attempts to answer is whether the original SDQ’s structure should be retained or should be refined to be used in this population. The availability of a culturally appropriate SDQ model could support the early detection of emotional and behavioral problems among refugee adolescents and support more targeted mental health interventions by schools, healthcare providers, and humanitarian organizations.

## Methods

This study is part of a larger project addressing the health needs of adolescent girls in Palestine refugee camps in the West Bank and Jordan. The broader project aimed to explore multiple dimensions of adolescent girls’ health, including sexual and reproductive health, mental well-being, and the social and environmental factors influencing their health. As part of this project, the Strengths and Difficulties Questionnaire (SDQ) was administered to assess emotional and behavioral well-being and to evaluate its psychometric properties in this understudied population [36,37]. The current study, which validates the SDQ, is based on data collected during the quantitative phase of the project. Given the scope of the research, male adolescents were not included in this study.

### Study setting

The study focused on adolescent girls aged 15–18 years residing in Palestinian refugee camps located in the West Bank, Palestine, and in Jordan. These camps were originally established after 1949, following the displacement of Palestinians from their homelands to other places in Palestine and neighboring countries. These refugee camps now host third- and fourth-generation refugees. There were 19 official refugee camps in the West Bank and 10 in Jordan [38].

### Sampling and recruitment

To achieve broad representativeness, data collection was undertaken in all 29 refugee camps across the two settings. A stratified random sampling approach was applied, with strata defined according to camp population size. Within each camp, a door-to-door “random walk” method was used to select households [39]. Trained women fieldworkers, either residents of the camps or familiar with camp contexts, carried out the recruitment and interviews.

Fieldworkers knocked on every nth door within the catchment area based on the “random walk” technique. Fieldworkers introduced themselves and the study and then asked if the household had adolescent girls between the ages of 15–18. If they did, they were eligible to be included in the sample. The fieldworker then obtained consent for participation in the study. Consents were obtained through a two-step process: initial approval from the mother, followed by consent from the adolescent girls. The eligibility criteria for this study included girls aged 15–18 years who were residing in a refugee camp in either the West Bank or Jordan at the time of the study, and who had experienced menarche. All individuals approached to participate in the study were informed that participation was entirely voluntary and that they could withdraw from the study at any time without any consequences. They were also assured that their decision to participate, decline participation, or withdraw would not affect their access to any services. Consent was obtained in two stages. First, informed consent was obtained from the mother or another adult guardian in the household. Second, informed consent was obtained from the adolescent girl herself. At both stages, participants were informed that they could decline participation altogether or choose not to answer any question they did not wish to answer.

See further details in S1 Appendix for the sample size calculation.

### Data collection and instruments

**Fig 1:**
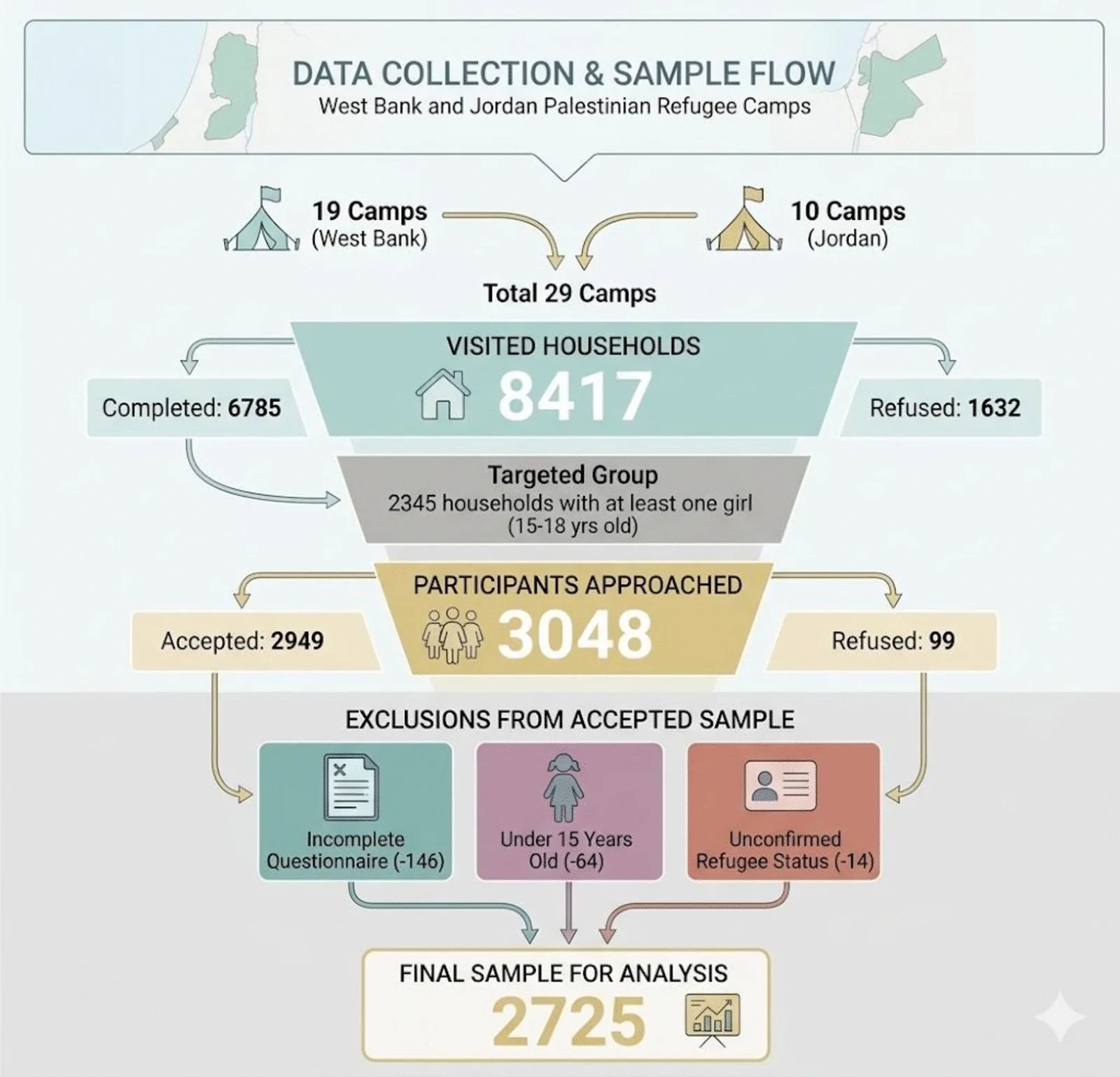
Data collection and sample flow.

Two structured survey tools were used. Data were collected by the fieldworker, who read out each question to the respondent and recorded the response on a tablet. Mothers responded to household roster questionnaire capturing demographic and socioeconomic data. Adolescent girls responded to a separate questionnaire covering individual demographics, socioeconomic status, general health, health-related behaviors, and mental health status, including the SDQ. After data were collected on the tablets, they were uploaded to a secure local server. Data collection was conducted between June and September 2019. Fig 1 shows the data collection and sample flow.

### SDQ description

The original SDQ is a 25-item tool that is used for evaluating five different dimensions: emotional symptoms, conduct problems, hyperactivity, peer problems, and pro-social behaviors. The Arabic version of the SDQ was validated by Alyahri and Goodman back in 2006 [40]. Each dimension is assessed via five statements using a 3-point Likert scale (not true, somewhat true, and certainly true). The items were phrased into a combination of positive and negatively directed statements [10,41]. The list of statements and their corresponding labels is presented in Table 1. The score for each dimension of the original SDQ is the result of the summation of the responses for the five items designated for that dimension. The range of the score of each dimension is from 0 to 10. The SDQ’s difficulties score is calculated by combining hyperactivity, emotional symptoms, conduct problems, and peer problems scores, ranging from 0 to 40. Given the conceptual difference of the pro-social score, it is not included in the total score calculation [41]. For this study, the items and the range of the possible score will be suggested based on the factor analysis results.

**Table 1:** List of SDQ statements and their labels.

Item No.	Item wording	Item label	Subscale
V1	I try to be nice to other people. I care about their feelings	Considerate	Prosocial
V2	I am restless, I cannot stay still for long	Restless	Hyperactivity/Inattention
V3	I get a lot of headaches, stomach aches, or sickness	Somatic	Emotional Symptoms
V4	I usually share with others (food, games, pens, etc.)	Shares	Prosocial
V5	I get very angry and often lose my temper	Tantrum/Tempers	Conduct Problems
V6	I am usually on my own. I generally play alone or keep to myself	Loner/Solitary	Peer Problems
V7	I usually do as I am told	Obey/Obedient	Conduct Problems
V8	I worry a lot	Worries	Emotional Symptoms
V9	I am helpful if someone is hurt, upset, or feeling ill	Caring	Prosocial
V10	I am constantly fidgeting or squirming	Fidgety	Hyperactivity/Inattention
V11	I have one good friend or more	Friend	Peer Problems
V12	I fight a lot. I can make other people do what I want	Fights	Conduct Problems
V13	I am often unhappy, downhearted, or tearful	Unhappy	Emotional Symptoms
V14	Other people my age generally like me	Popular	Peer Problems
V15	I am easily distracted; I find it difficult to concentrate	Distractible	Hyperactivity/Inattention
V16	I am nervous in new situations. I easily lose confidence	Clingy	Emotional Symptoms
V17	I am kind to younger children	Kind	Prosocial
V18	I am often accused of lying or cheating	Lies	Conduct Problems
V19	Other children or young people pick on me or bully me	Bullied	Peer Problems
V20	I often volunteer to help others (parents, teachers, children)	Help out/Helps	Prosocial
V21	I think before I do things	Reflective	Hyperactivity/Inattention
V22	I take things that are not mine from home, school, or elsewhere	Steals	Conduct Problems
V23	I get on better with adults than with people my own age	Oldbest/Adults	Peer Problems
V24	I have many fears; I am easily scared	Afraid/Fears	Emotional Symptoms
V25	I finish the work I’m doing. My attention is good	Attention/Persistent	Hyperactivity/Inattention

### Statistical analysis

The psychometric properties of the original SDQ and other suggested alternatives were tested in this study using various statistical analyses. First, construct validity for the original SDQ was tested through confirmatory factor analysis (CFA). Then, exploratory factor analysis (EFA) was used to evaluate the latent factorial structure for various suggested alternatives, followed by CFA to provide additional evaluation of each suggested alternative. Second, further validation tests were conducted on the final suggested alternative scale. Convergent, discriminant, and criterion validity were assessed to determine whether the tool relates to other similar or distinct constructs. Third, the internal consistency and reliability were assessed using multiple tests. Based on the results of the EFA and CFA, the new difficulties scale’s score will be calculated using the remaining items (excluding any prosocial items) of the suggested alternative with the best structure. The following sections describe each analysis in detail. SPSS 30 and JASP 0.19.3 software were used to conduct the analyses.

### Construct validity

Construct validity is the extent to which the scale can actually measure what it is intended to measure [42]. EFA and CFA were used to evaluate the construct validity of the original SDQ and the suggested alternative [42]. A sample of at least 1,000 is considered excellent for conducting factor analysis [43].

First, we conducted CFA to check the factorial structure of the original SDQ scale and that of other suggested versions. To evaluate the goodness of fit of the CFA model, we calculated the Chi-Squared (χ^2^) statistic, which should have a non-significant p-value (>0.05) and a χ^2^ /Degree of freedom (*df*) ratio ≤2. Furthermore, we checked several indices and tests, including the root mean square error of approximation (RMSEA; ≤ 0.06), Tucker–Lewis index (TLI; > 0.9), comparative fit index (CFI; > 0.9) [44], and the standardized root mean square residual (SRMR; < 0.08) [44].

We then conducted multiple EFA analyses to check various alternative versions of the underlying factorial structure to achieve models with good fit. EFA was preferred over principal component analysis (PCA), given the nature of their purposes. EFA considers the existence of underlying latent factors, while PCA primarily concerns item reduction [45].

We conducted the Kaiser–Meyer–Olkin (KMO) test to assess the sampling adequacy of the EFA. Values >0.8 indicate adequate sampling for EFA, while values between 0.7 and 0.8 are good [46,47]. A statistically significant Bartlett’s test of sphericity (p-value *<*0.05) was used to confirm that the variables were correlated sufficiently to conduct EFA [46]. Multicollinearity was checked by referring to the determinant value (>0.00001) [48]. Variables with anti-image correlation values greater than 0.50 were considered suitable for factor analysis [48].

To determine the number of factors, we used parallel analysis and a scree plot [42,44]. Factors with loadings <0.40 were eliminated [42,44]. We conducted the EFA and CFA iteratively, each time eliminating items that failed to meet the loading threshold (<0.40) in either the EFA or CFA. Subsequent re-evaluation of the factorial structure was conducted. After each round of item elimination, parallel analysis, scree plot inspection, EFA, and CFA were conducted to confirm the optimal number of factors and the stability of the factorial structure [49]. This process was repeated until a psychometrically sound model was obtained. The final proposed model was critically evaluated in terms of its theoretical and cultural foundations to evaluate whether the proposed alternative, comprising the retained items and any variations in their factor loadings, can be theoretically justified and properly interpreted within cultural values, norms, and lived experiences of adolescent Palestinian refugee girls.

### Convergent and discriminant validity

Convergent validity can be evaluated based on the results of the correlation with another scale that measures a similar construct [42,44]. In this study, we evaluate convergent validity through testing correlation with the WHO-5 well-being scale. The average variance extracted (AVE) was calculated to assess how much variance in the observed items is explained by the latent factors. Correlations should ideally be at least 0.5 [50], while standardized factor loadings should be at least 0.40. In the event of a finding of poor or unsatisfactory convergent validity, discriminant validity will not be assessed [50].

### Criterion validity

Criterion concurrent validity assesses if the tool has a strong association with a criterion tool measured at the same time [42,44]. The criterion validity of the SDQ difficulties score (for the newly suggested alternative) is evaluated using the receiver operating characteristic (ROC) curve, as its structure is tested with a gold-standard outcome [42,44]. If the area under the curve (AUC) is at least 0.70, then it is acceptable, while an AUC higher than 0.8 is good [51]. The ROC analysis was also used to suggest possible cut-off points for the difficulty scale, relying on Youden’s index. The variables used for this purpose are:

1) Self-reported diagnosis of a psychological or mental illness was used to serve as an external ‘gold standard’ based on a previous diagnosis. A similar approach was reported in previous studies in which the SDQ validity was evaluated based on the strength of its correlation with diagnosed psychopathology and psychosocial issues [52–55]. We assessed the criterion validity using the question “Have you been diagnosed with a psychological/ mental illness condition?” with ‘Yes’ or ‘No’ as the possible responses.
2) Life satisfaction reflects a broader subjective evaluation of adolescents’ lives [56]. Adolescents with low life satisfaction were predicted to be more likely to have psychological problems. On the other hand, higher satisfaction was associated with fewer emotional and behavioral problems among adolescents [57]. SDQ was also found to be associated with school children and adolescents’ life satisfaction [56]. Therefore, life satisfaction is a relevant external criterion for assessing the validity of mental health screening tools such as the SDQ. It can be a suitable indicator for concurrent validity, as a strong correlation between these two measures can support the SDQ as a measure of adolescents’ overall well-being. It was assessed using the question “To what extent are you satisfied with your life?” with responses ranging between ‘Very bad’ to ‘Very good’.
3) Self-perceived difficulties: This variable mirrors what SDQ measures; therefore, it would be a good indicator of the SDQ’s validity. It was assessed by the question “Overall, do you think that you have difficulties in any of the following areas: emotions, concentration, behavior, or being able to get on with other people?” Girls had to choose one of the following answers to respond to this question: ‘No’, ‘Minor difficulties’, Definite difficulties’, or ‘Severe difficulties.
4) SDQ was reported to be valid for measuring concurrent mental health issues [52–55], such as depression [52,55,58]. Given that the WHO-5 well-being score below 50 was used to identify ‘Low well-being’, and scoring 28 or lower can indicate a risk of depression [59], we assumed that the association between the SDQ and the WHO-5 (as an indicator for risk of depression) might suggest that the SDQ is suitable for accurately identifying individuals with difficulties.

### Reliability

To measure the reliability of the suggested versions, we used Cronbach’s α [44], Guttman’s λ2 and McDonald’s ω. This was based on the suggested superiority of Guttman’s λ2 and McDonald’s ω over α [60,61]. Other tests, like average inter-item correlations [62], item-to-total correlations [63], and split-half reliability [64] were conducted.

### Ethical considerations

The study received ethical approval from the Research Ethics Committee of the Institute of Community and Public Health at Birzeit University, Palestine (approval no. 171114), and from the Regional Committee for Medical and Health Research Ethics (REC), South-East Norway (reference no. 2018/2206). Both committees also approved subsequent amendments. In Norway, the amendment was approved under the original reference number (2018/2206). At Birzeit University, the original approval was extended through an addendum (submission reference no. 201129), with all conditions of the initial approval remaining applicable. Before participation, written informed consent was obtained from the girls’ caregivers and from the adolescent girls themselves.

### Inclusivity in global research

The study was conducted in close collaboration with local researchers, institutions, and community representatives in Palestine and Jordan. Approvals were obtained from the relevant institutional and community authorities before implementation, and the study design was informed by consultations with community stakeholders and an exploratory qualitative phase. Participant-facing materials were prepared in Arabic and adapted to the local context to ensure informed participation. Additional information regarding the ethical, cultural, and scientific considerations specific to inclusivity in global research is provided in (S2 Checklist and S3 Checklist).

## Results

This study included 2,828 adolescent girls living in Palestine refugee camps in the West Bank and Jordan. Of these, 2,725 participants were included in the final analysis based on completion of the Strengths and Difficulties Questionnaire (SDQ). The mean age of the girls was 16.77 (Standard deviation [SD] 1.14), with ages ranging from 15 to 18 years. The majority of them were single (93.2%), with only 185 (6.8%) having ever been engaged or married. Participants were almost equally distributed between the West Bank (49.7%) and Jordan (50.3%). Most girls lived in urban camp communities (73.5%), while 22.9% lived in rural communities and 3.7% in Bedouin communities. Among those with valid responses, the majority lived in nuclear families (95.3%), whereas 4.7% lived in extended families. Regarding household standard of living, 40.2% were classified as having a low standard of living, 33.9% as moderate, and 25.9% as high. Further details are shown in Table 2.

**Table 2:** Characteristics of the adolescent girls who participated in this study.

Variable	Categories	N (%)
**Age (years)**	<16	852 (31.3%)
	16 – < 17	673 (24.7%)
	≥17	1,200 (44.0%)
**Marital status**	Single	2,540 (93.2%)
	Engaged	120 (4.4%)
	Married	60 (2.2%)
	Separated	5 (0.2%)
**Registered as a Palestinian refugee (UNRWA)**	No	37 (1.4%)
	Yes	2,688 (98.6%)
**Camp location (country)**	West Bank	1,354 (49.7%)
	Jordan	1,371 (50.3%)
**Nature of camp community**	Urban	2,002 (73.5%)
	Rural	623 (22.9%)
	Bedouin	100 (3.7%)
**Family type**	Nuclear family (parents and siblings only)	2,518 (95.3%)
	Extended family	123 (4.7%)
**Household standard of living index**	Low (≤8)	1,062 (40.2%)
	Medium (9–11)	895 (33.9%)
	High (≥12)	684 (25.9%)
**Do you feel that your material needs are met?**	Strongly disagree	40 (1.5%)
	Disagree	175 (6.4%)
	Neutral	270 (9.9%)
	Agree	1,802 (66.2%)
	Strongly agree	435 (16.0%)
**Perceived economic status compared to others**	Very bad	68 (2.5%)
	Bad	282 (10.3%)
	Fair	1,388 (50.9%)
	Good	734 (26.9%)
	Very good	253 (9.3%)

### Validity

#### Construct validity: CFA results of the SDQ original scale.

**Fig 2:**
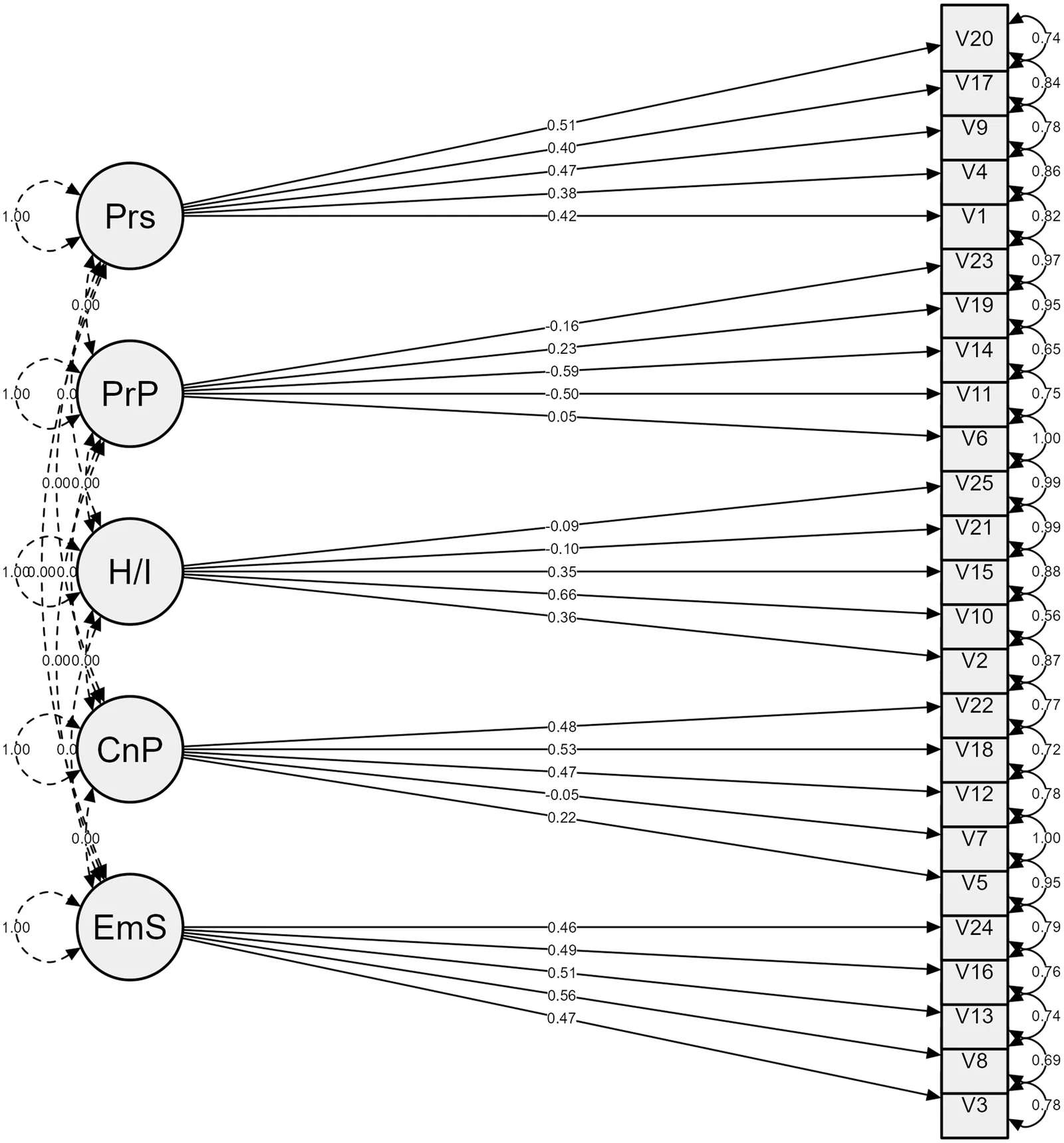
The model plot for the original SDQ. Prs, Prosocial; PrP, Peer Problems; H/I, Hyperactivity/Inattention; CnP, Conduct Problems; EmS, Emotional Symptoms.

Table 3 presents the results of various model fit indices for the original 5-factor model of the SDQ. The original 5-factor model has a poor fit, with CFI and TLI values below 0.4 and an RMSEA of 0.081, while the SRMR equals 0.110. Fig 2 shows the standardized factor loadings of the original SDQ. Several items had low loadings. The items ‘Tantrum’ and ‘Obeys/Obedient’ in Factor 2, ‘Reflective’ and ‘Attention/Persistent’ in Factor 3, and ‘Loner/solitary’, ‘Bullied’, and ‘Oldbest/Adults’ in Factor 4 had standardized loadings less than 0.25. Furthermore, items ‘restless’, ‘shares’, and ‘distractible’ had factor loadings below 0.40 on their respective factors, whereas all other loadings were above 0.40.

**Table 3:** Model fit measures for the original SDQ and the suggested alternatives.

	Original	Suggested alternatives	
*Fit indices*	5-factor	4-factor	3-factor
Chi-square test (Χ^2^)	4946.568 *df = 275, < 0.001*	778 *df = 146, < 0.001*	χ2=358.2, *df=78, <0.001*
Comparative Fit Index (CFI)	0.397	0.896	0.942
Tucker-Lewis Index (TLI)	0.342	0.879	0.930
Root mean square error of approximation (RMSEA)	0.081	0.040	0.034
RMSEA 90% CI lower bound	0.079	0.038	0.030
RMSEA 90% CI upper bound	0.083	0.043	0.038
RMSEA p-value	<0.001	1.000	1.000
Standardized root mean square residual (SRMR)	0.110	0.039	0.033
Average variance extracted (AVE)			
Factor 1: Emotional Symptoms	0.249	0.246	0.233
Factor 2: Conduct Problems	0.148	0.207	0.173
Factor 3: Hyperactivity/Inattention	0.162	0.347	0.342
Factor 4: Peer Problems	0.109	0.337	
Factor 5: Pro-social	0.188		

When we conducted the exploratory factor analysis (EFA) and manually selected items to load onto five factors, the resulting structure differed from the original SDQ scale (Table 4). The original ‘emotional symptoms’ subscale included ‘Somatic’, ‘Worries’, ‘Unhappy’, ‘Clingy’, and ‘Afraid’. However, additional items— ‘Tantrum’ from ‘Conduct Problems’, Loner’ from ‘peer problems’, and ‘Distractable’ from ‘hyperactivity/inattention’—also loaded onto this factor. The item ‘Reflective’, originally part of ‘Hyperactivity/Inattention’, loaded onto the ‘Prosocial’ subscale, whereas ‘Shares’ and ’Caring’ were excluded due to loadings below 0.40, resulting in a four-item ‘prosocial’ factor. ‘Fidgety’ and ‘Restless’ were loaded onto the ‘Hyperactivity/Inattention’ factor. The item ’Bullied’ loaded onto the ‘Conduct Problems’ factor, alongside ‘Steals’ and ‘Lies’.

**Table 4:** Comparison between the original SDQ and EFA.

	Original SDQ Subscale	New EFA Factor	
Item (Code)		Factors	Factor Loading
V3. Somatic	Emotional Symptoms	Factor 1 (Emotion)	0.439
V8. Worries	Emotional Symptoms	Factor 1 (Emotion)	0.542
V13. Unhappy	Emotional Symptoms	Factor 1 (Emotion)	0.644
V16. Clingy	Emotional Symptoms	Factor 1 (Emotion)	0.438
V24. Afraid/Fears	Emotional Symptoms	Factor 1 (Emotion)	0.410
V5. Tantrum/Tempers	Conduct Problems	Factor 1 (Emotion)	0.453
V6. Loner/Solitary	Peer Problems	Factor 1 (Emotion)	0.496
V15. Distractible	Hyperactivity/Inattention	Factor 1 (Emotion)	0.467
V21. Reflective	Hyperactivity/Inattention	Factor 2 (Prosocial)	0.480
V20. Help out/Helps	Prosocial	Factor 2 (Prosocial)	0.457
V17. Kind	Prosocial	Factor 2 (Prosocial)	0.434
V1. Considerate	Prosocial	Factor 2 (Prosocial)	0.419
V19. Bullied	Peer Problems	Factor 3 (Conduct)	0.666
V22. Steals	Conduct Problems	Factor 3 (Conduct)	0.549
V18. Lies	Conduct Problems	Factor 3 (Conduct)	0.513
V10. Fidgety	Hyperactivity/Inattention	Factor 4 (Hyperactivity)	0.519
V2. Restless	Hyperactivity/Inattention	Factor 4 (Hyperactivity)	0.493
V14. Popular	Peer Problems	Factor 5 (Peer Relations)	0.517
V4. Shares	Prosocial	Dropped	< 0.40
V7. Obey/Obedient	Conduct Problems	Dropped	< 0.40
V9. Caring	Prosocial	Dropped	< 0.40
V11. Friend	Peer Problems	Dropped	< 0.40
V12. Fights	Conduct Problems	Dropped	< 0.40
V23. Oldbest/Adults	Peer Problems	Dropped	< 0.40
V25. Attention/Persistent	Hyperactivity/Inattention	Dropped	< 0.40

### Construct validity: Psychometric analysis results for suggested SDQ alternatives

#### 4-Factor Solution.

**Fig 3:**
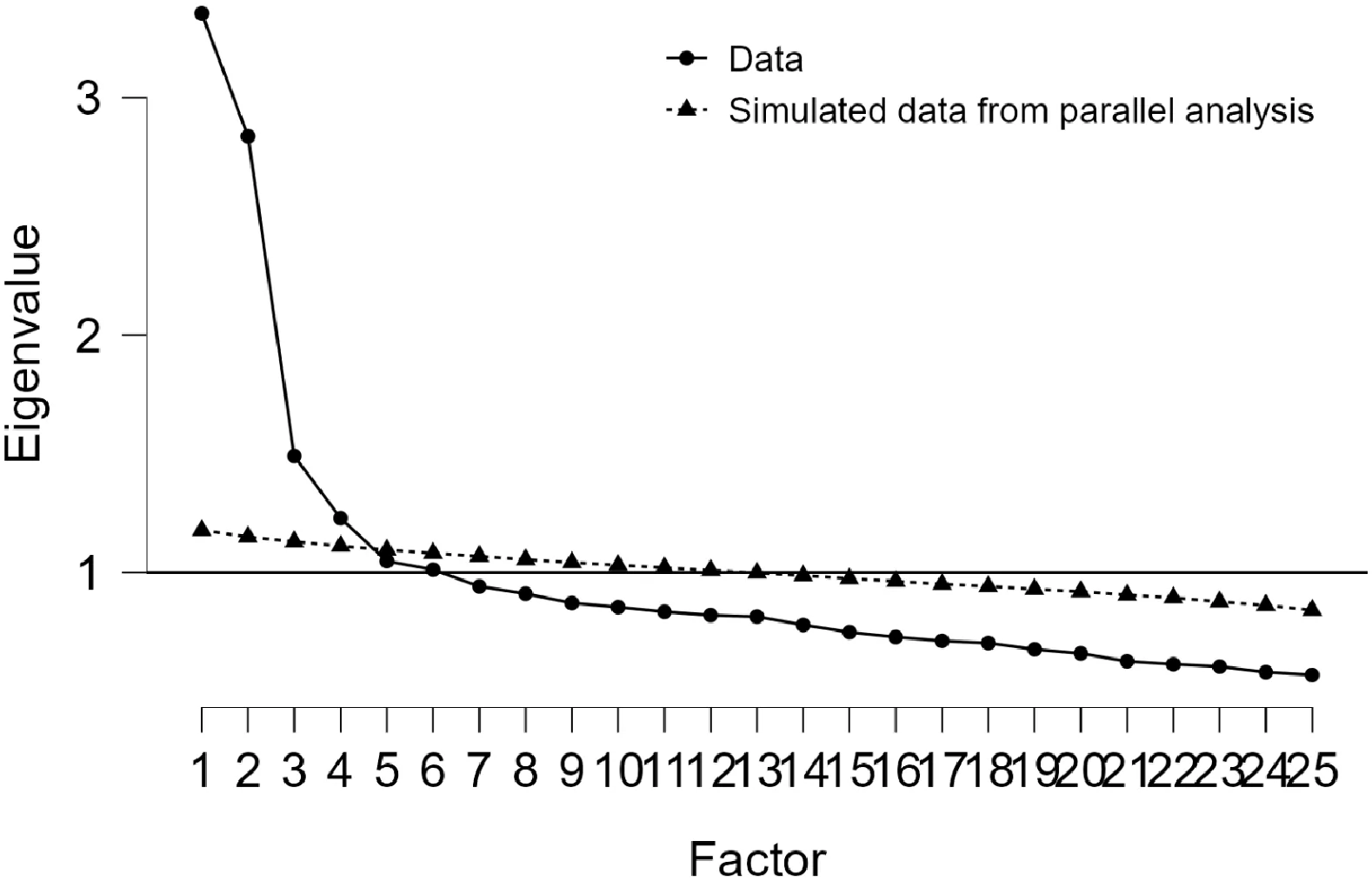
Scree plot showing the suggested number of factors to be retained.

Table 5 presents the rotated factor loadings for the 25 items that belong to the four-factor solution. Based on the Scree plot shown in Fig 3, one of the suggested alternatives for the SDQ scales was the 4-factor solution. EFA was conducted using the principal axis factoring method of extraction. The 4-factor solution model indicates that only 19 of the 25 SDQ items should be retained. ‘Considerate’, ‘Shares’, ‘Obeys/Obedient’, ‘Friend’, ‘Fights’, and ‘Oldbest/Adults’ were removed as they had loadings less than 0.40. The overall KMO statistic was 0.808, while Bartlett’s test of sphericity was significant (χ2 = 6247, p < 0.001). The anti-image correlation matrix diagonal values were all greater than 0.688. The sample did not have multicollinearity based on the determinant value (0.095).

**Table 5:** The characteristics of the 4-Factor solution.

Factor	Item	EFA Loading	IRC	AIC	α	ω	λ2	Split-half
**Factor 1: Emotional Symptoms**	V13. Unhappy	0.638	0.434	0.245	0.721	0.722	0.722	0.739
	V8. Worries	0.523	0.457					
	V6. Loner/Solitary	0.509	0.378					
	V5. Tantrum/Tempers	0.482	0.443					
	V16. Clingy	0.467	0.442					
	V15. Distractible	0.439	0.398					
	V24. Afraid/Fears	0.424	0.344					
	V3. Somatic	0.414	0.400					
**Factor 2: Prosocial**	V20. Help out/Helps	0.539	0.394	0.209	0.611	0.612	0.613	0.617
	V21. Reflective	0.477	0.306					
	V25. Attention/Persistent	0.472	0.369					
	V14. Popular	0.438	0.348					
	V17. Kind	0.416	0.326					
	V9. Caring	0.403	0.324					
**Factor 3: Conduct Problems**	V19. Bullied	0.668	0.449	0.322	0.594	0.600	0.596	0.469
	V18. Lies	0.530	0.414					
	V22. Steals	0.469	0.358					
**Factor 4: Hyper-activity/** **Inattention**	V2. Restless	0.535	0.236	0.236	0.381	N/A	0.381	0.381
	V10. Fidgety	0.436	0.236					

KMO = 0.808, Bartlet test: Χ² = 6247, Df = 171, p-value < 0.001; Applied rotation method is Promax; AIC, Average inter-item correlation; * Standardized loadings.

As shown in Table 5, Factor 1, emotional distress, included eight items that explained about 13% of the variance, with factor loadings ranging from 0.414 to 0.638. Factor 2, prosocial, included six items that explained 8% of the total variance. Factor 2 loadings ranged between 0.403 and 0.539, while factor 3 items’ loadings ranged between 0.469 and 0.668. This factor is composed of three items and explains only 3.7% of the total variance. The final factor consists of 2 items, explaining only 2.4% of the total variance, with factor loadings of 0.535 and 0.436.

**Fig 4:**
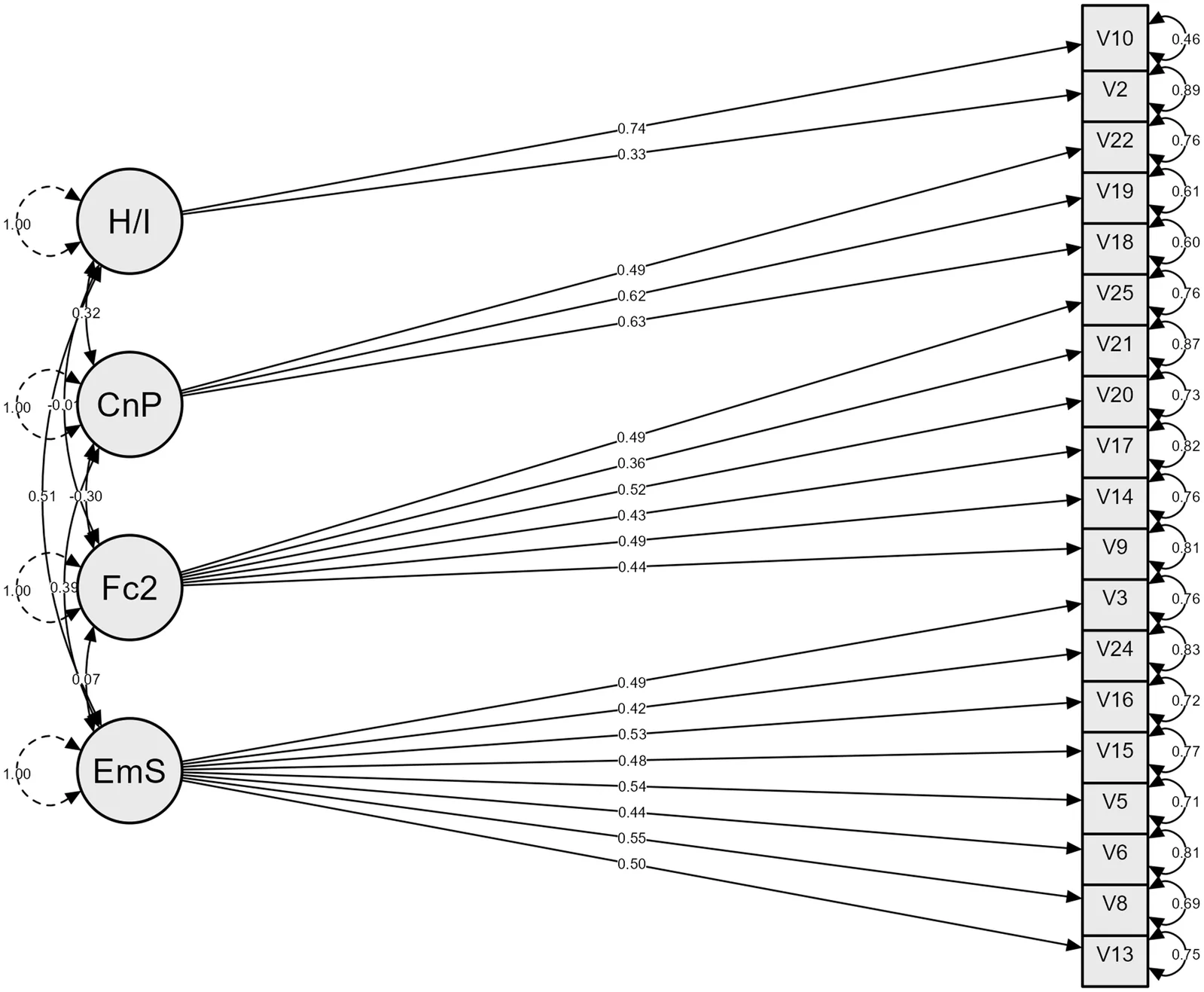
Model plot for the 4-factor solution.

Fig 4 shows that the standardized loadings for factor 1 were between 0.42 and 0.58, while for factor 2 the loadings ranged from 0.36 to 0.52. For factors 3 and 4, the loadings ranged from 0.49 to 0.63 and from 0.33 to 0.74, respectively. The χ2 statistic was significant (χ2 = 778, df = 146, < 0.001). The χ2:*df* ratio was 5.3. Other goodness-of-fit tests indicated a good fit for the model. RMSEA was 0.040, while SRMR was 0.039. The TLI and CFI values were slightly below 0.90 (CFI = 0.896 and TLI = 0.879).

#### 3-Factor solution.

**Fig 5:**
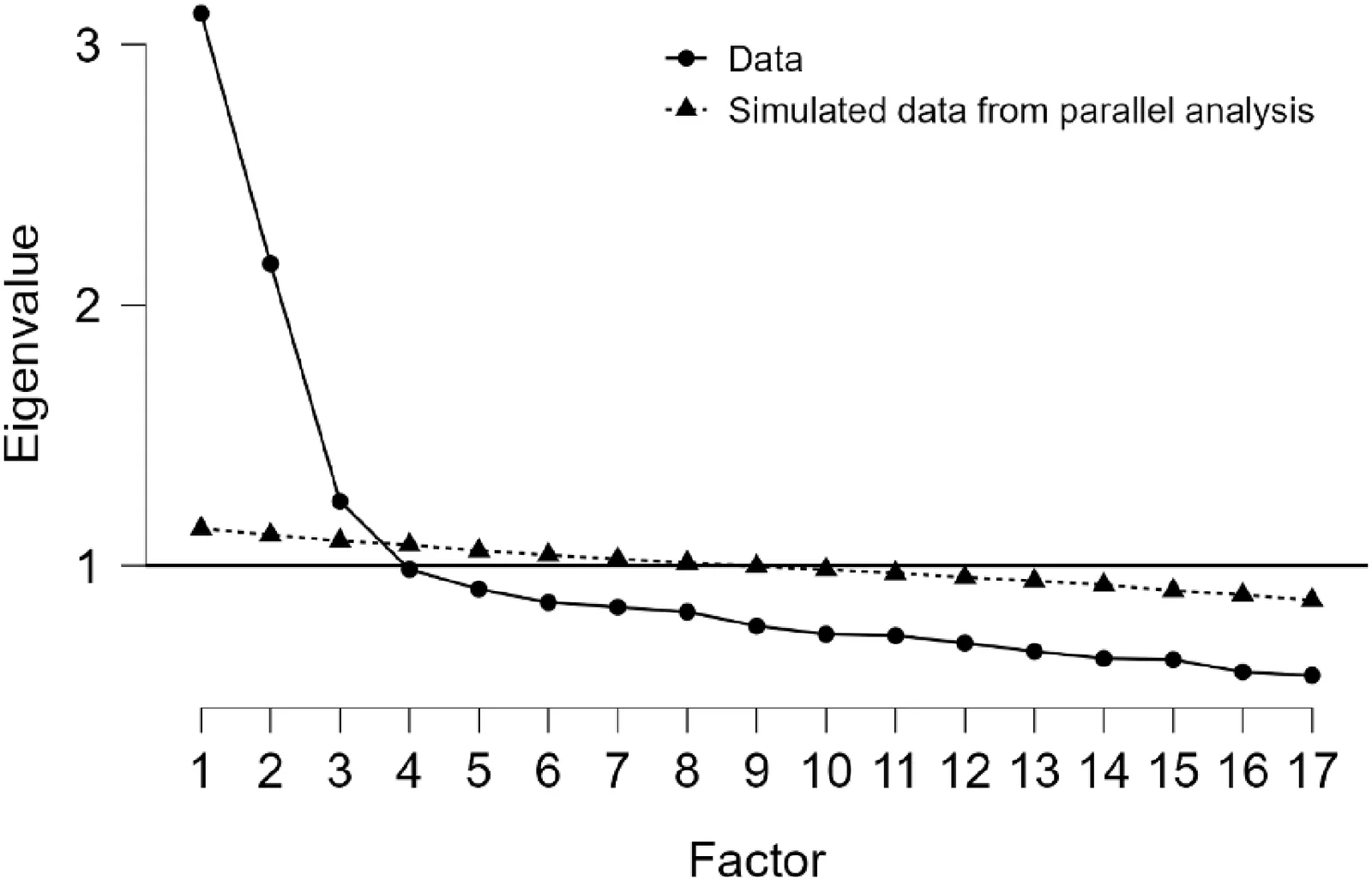
Scree plot showing the suggested number of factors to be retained after eliminating items based on CFA of the 4-Factor model.

The three-factor solution demonstrated adequate sampling adequacy (KMO = 0.792) and a significant Bartlett’s test of sphericity (Χ² = 4797, df = 105, p < .001), supporting the factorability of the data. Based on the CFA (for the 4-factor solution), the items ‘Restless’ and ‘Reflective’ had loadings less than 0.40. After eliminating both items, parallel analysis and Scree plot showed that we should retain three factors (Fig 5). After the EFA was run again, items ‘Fidgety’ and ‘Afraid/Fears’ needed to be dropped, retaining 15 items out of the original 25 items in three factors.

Table 6 presents the rotated factor loadings for the 15 items retained from the previous steps. Based on the parallel analysis, the 15 items loaded into three factors: ‘Emotional Symptoms’, ‘Prosocial’, and ‘Conduct Problems’. ‘Considerate,’ ‘Restless,’ ‘Shares,’ ‘Obeys/Obedient,’ ‘Fidgety,’ ‘Friend,’ ‘Fights,’ ‘Reflective,’ ‘Oldbest/Adults,’ and ‘Afraid/Fears’ were removed as they had loadings less than 0.40 in EFA and CFA. The overall KMO statistic was 0.792, while Bartlett’s test of sphericity was significant (Χ² = 4797, p-value < 0.001). The anti-image correlation matrix diagonal values were all greater than 0.723. The determinant value was 0.166, indicating no multicollinearity.

**Table 6:** The characteristics of the 3-Factor solution.

Factor	Item	EFA Loading	IRC	AIC	α	ω	λ2	Split-half
**Factor 1: Emotional Distress**	V5. Tantrum/Tempers	0.594	0.463	0.257	0.708	0.709	0.709	0.727
	V13. Unhappy	0.527	0.424					
	V8. Worries	0.517	0.436					
	V16. Clingy	0.490	0.431					
	V3. Somatic	0.477	0.400					
	V6. Loner/Solitary	0.468	0.378					
	V15. Distractible	0.465	0.390					
**Factor 2: Prosocial**	V20. Help out/Helps	0.607	0.386	0.221	0.584	0.586	0.586	0.593
	V14. Popular	0.467	0.354					
	V9. Caring	0.455	0.335					
	V25. Attention/Persistent	0.430	0.332					
	V17. Kind	0.405	0.304					
**Factor 3: Conduct**	V19. Bullied	0.669	0.449	0.322	0.594	0.600	0.596	0.469
	V18. Lies	0.608	0.414					
	V22. Steals	0.470	0.358					

KMO = 0.792, Bartlet test: Χ² = 4797, *df* = 105 p-value < 0.001; Applied rotation method is promax; AIC, Average inter-item correlation; After evaluating the psychometric properties, the total score for the difficulties scales was calculated. All the remaining items reported in this table were used to calculate the difficulties score. Scores ranged between 0 and 20 with a mean of 7.08 (SD3.8). Cronbach’s alpha, Guttman λ2, and MacDonald’s ω were higher than 0.710.

Factor 1, ‘Emotional Symptoms’, included seven items that explained about 13.7% of the variance, with factor loadings ranging from 0.465 to 0.594. Factor 2, ‘Prosocial’, included five items that explained 9.5% of the total variance. Factor 2 loadings ranged between 0.405 and 0.607, while factor 3 (Conduct Problems) items’ loadings ranged between 0.470 and 0.669. This factor is composed of three items, explaining only 3.7% of the total variance.

**Fig 6:**
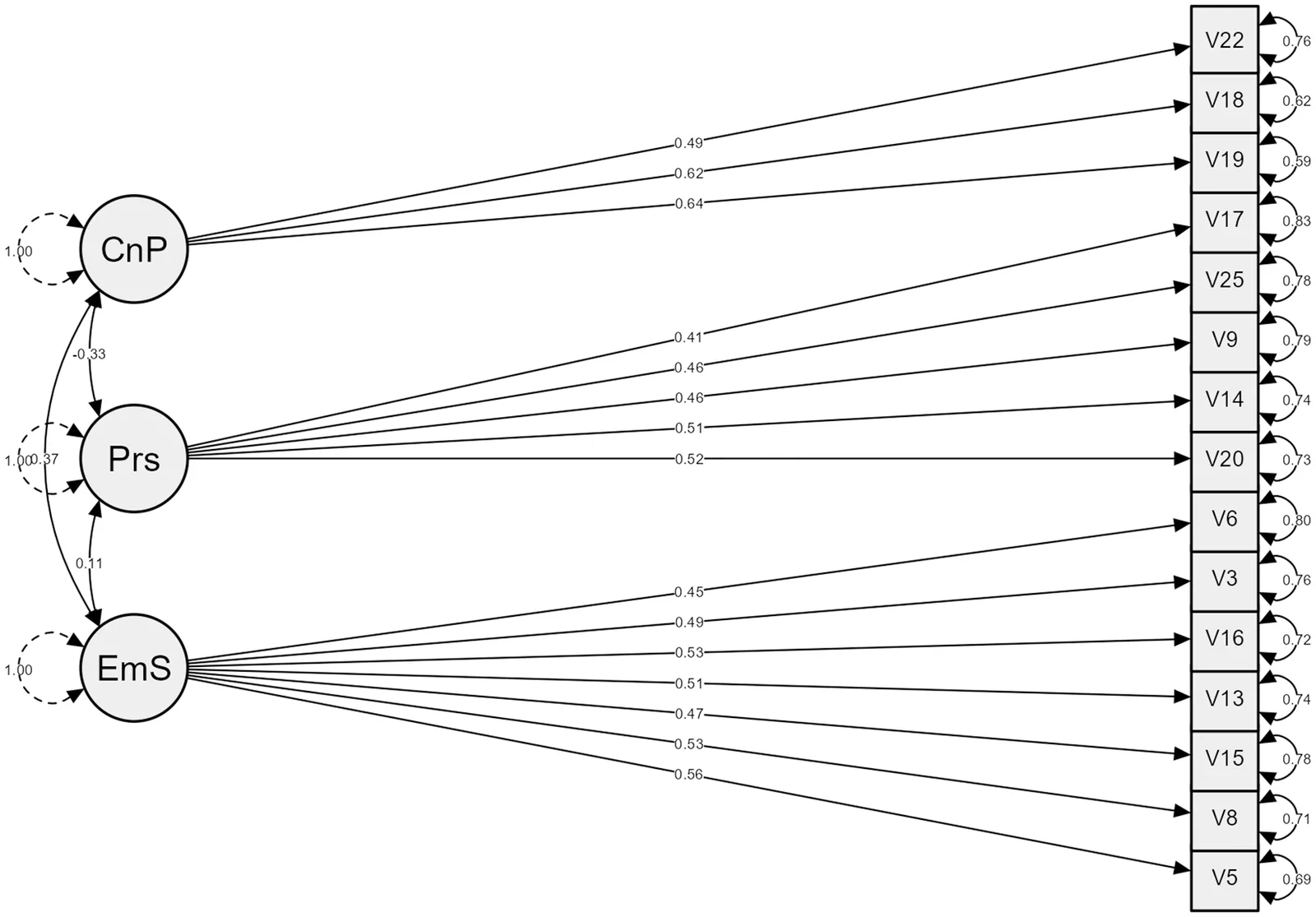
Model plot after removing additional items.

Fig 6 shows that the standardized loadings for factor 1 ranged from 0.47 to 0.56, while those for factor 2 ranged from 0.41 to 0.52. For factor 3, the loadings ranged from 0.49 to 0.64. The χ2 statistic was significant (χ2 = 358.2, df = 78, < 0.001). The χ2:*df* ratio was 4.6. Other goodness-of-fit tests indicated a good fit for the model. RMSEA was 0.034, while SRMR was 0.033. The TLI and CFI values were slightly below 0.95 (CFI = 0.942 and TLI = 0.930). See Table 3. Based on the results, the 3-factor solution is found to have the best structure compared to the other alternatives, and it will be used to calculate the difficulties score.

### Convergent and discriminant validity

The values of AVE were 0.233, 0.173, and 0.342 for factor 1, factor 2, and Factor 3 of the 3-factor alternative, respectively. The AVE values were lower than 0.50 for all factors of the other tested SDQ models (Table 2). The correlation coefficient between the ‘difficulties’ score and the WHO-5 well-being was −0.245. Given the low correlation, we do not need to test for discriminant validity.

### Criterion validity

**Fig 7:**
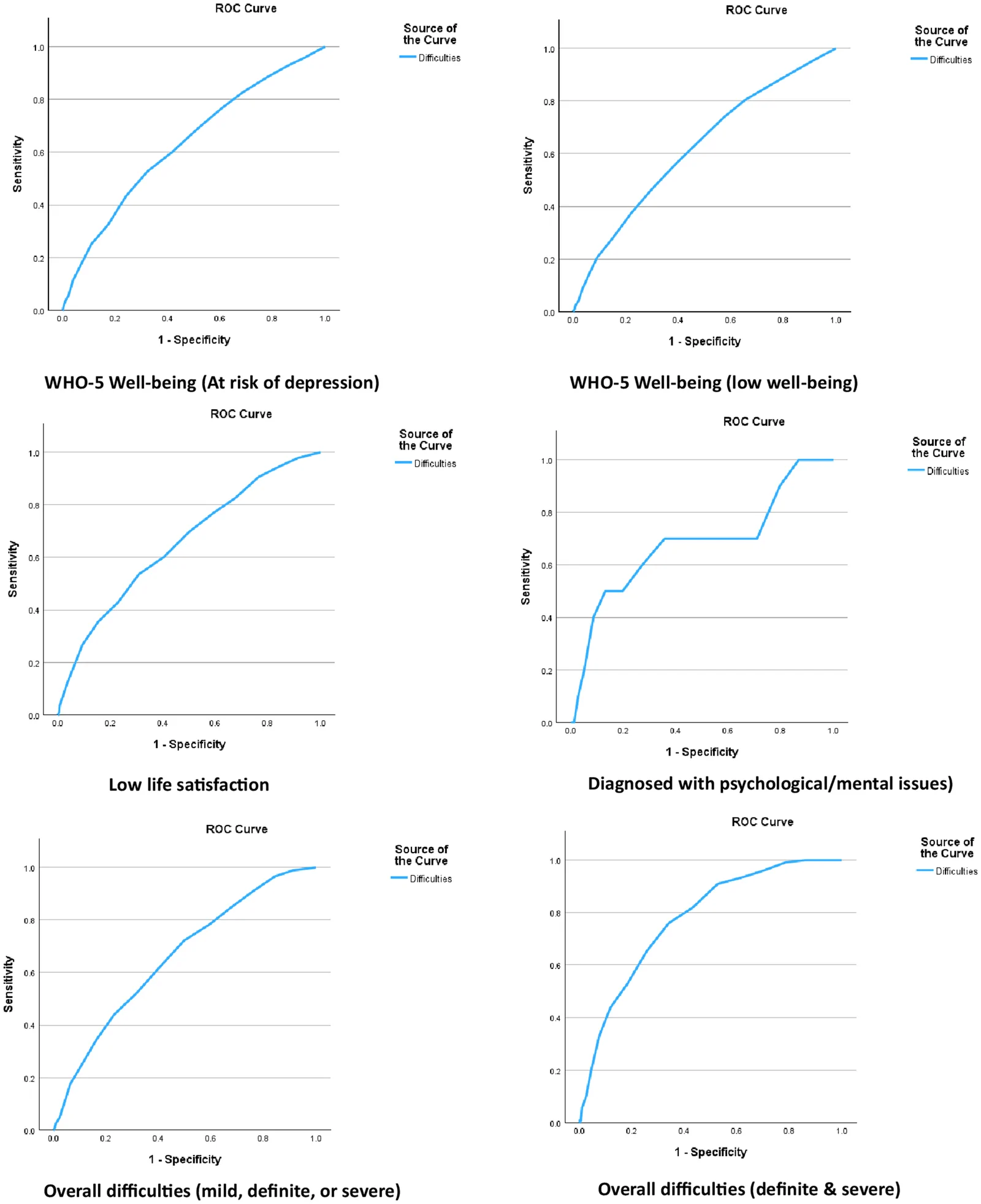
ROC curves for the variables used to test criterion concurrent validity.

The ROC curve analysis showed that all AUC values were poor (below 0.70) except for the overall difficulties (definite and severe), which was acceptable (0.776; Fig 7). The most common cut-off point for the scale is 8 or 9. The highest cut-off was based on the psychological/ mental issue diagnosis, which is 10 or 11. Further details are available in Table 7.

**Table 7:** The results of the ROC curve analysis for the difficulties scale across multiple validating variables.

Validation Variable	AUC	Evaluation	Overall model quality (<0.5)	Max Youden Index	Sensitivity at Max Youden	Specificity at Max Youden	Suggested Single Cut-off ++
**WHO-5 Wellbeing (at depression risk)**	0.633	Poor	0.60	0.204	0.529	0.675	**8.5** **8 or 9**
**WHO-5 Wellbeing (Low-wellbeing)**	0.621	poor	0.60	0.171	0.558	0.587	**7.5** **7 or 8**
**Life satisfaction**	0.654	poor	0.63	0.227	0.535	0.693	**8.5** **8 or 9**
**Psychological/ mental health issue**	0.680	Moderate	0.49	0.368	0.50	0.869	**11.5** **10 or 11**
**Overall difficulties (definite & severe)**	0.776	Acceptable	0.74	0.419	0.760	0.659	**8.5** **8 or 9**
**Overall difficulties (mild, definite, & severe)**	0.657	poor	0.63	0.224	0.722	0.502	**6.5** **6 or 7**

**++** The averages of two consecutive ordered observed test values; ROC, Receiver operating characteristic; AUC, Area under curve.

### Reliability

In the 4-factor solution, the average inter-item correlation for all factors ranged between 0.209 and 0.322. For the 4-Factor solution, item-to-total rest correlations were all above 0.324 for Factors 1, 2, & 3, while they were 0.236 for both items in Factor 4.

Internal consistency varied across factors in the 3-factor solution. Factor 1 (Emotional Symptoms) showed the strongest reliability (α = 0.708, ω = 0.709, λ2 = 0.709, split-half = 0.727, AIC = 0.257), with item-to-total correlations ranging from 0.378 to 0.436, indicating items are consistently aligned with the latent factor. Factor 2 (prosocial) demonstrated weaker reliability (α = 0.584, ω = 0.590, λ2 = 0.590, split-half = 0.590, AIC = 0.221), and all items had item-to-total correlations below 0.40 (IRC = 0.304–.0386), suggesting only modest internal coherence. Factor 3 (Conduct problems) had a borderline reliability (α = 0.594, ω = 0.600, λ2 = 0.600, split-half = .47, AIC = .32), with item-to-total correlations ranging from 0.358 to 0.449. This implies that low reliability estimates were partly attributable to the small number of items (k = 3). The Prosocial and Conduct scales require further refinement or expansion to enhance their internal consistency. Cronbach’s alpha for the composite difficulties scale (based on the 3-factor solution) was 0.71.

## Discussion

This study tested the psychometric properties of the original SDQ scale to evaluate whether it is valid and reliable for use among adolescent Palestinian refugee girls in the West Bank and Jordan. The results indicate that the original 5-factor SDQ has poor fit parameters. Therefore, this study suggested an alternative version of the SDQ based on the EFA and CFA analyses. The 3-factor model, which comprises 15 items, had acceptable fit indices. Besides the number of items and factors, several items in the newly suggested alternative loaded onto factors other than the ones they were designated to in the original SDQ. The 3-factor alternative showed poor convergent validity and acceptable criterion concurrent validity. The reliability tests were also acceptable for emotional symptoms (factor 1), while they were borderline acceptable for prosocial (factor 2) and conduct problems (factor 3).

### Construct validity

#### Original SDQ.

The CFA showed that among Palestinian refugee dwellers, the original SDQ structure had poor fit indices. None of the fit indices used to assess model fit met the threshold to consider the original SDQ structure a good fit. The Chi-square test was statistically significant, indicating a poor fit. Since the Chi-square test is sensitive to sample size, a larger sample size might lead to a decreased p-value [65], which might explain why we had a statistically significant Chi-square test for the original 5-factor model and the 4-and 3-factor alternative models. Other indices, such as TLI and CFI values, were lower than the recommended thresholds for both the original 5-factor and the alternative 4-factor models, where TLI and CFI were below 0.40 and 0.90, respectively. According to the literature, both indices should be at least 0.90 to indicate an acceptable model fit [44]. This was achieved in the alternative 3-factor model, as both TLI and CFI were ≥0.93, which indicates an acceptable fit and is close to 0.95, which some would consider the threshold for a good model fit [44,66]. Additionally, both RMSEA and SRMR were higher than the recommended cutoffs of 0.06 and 0.1, respectively [44,66] for the original 5-factor model, but not for the 4- and 3-factor alternatives. However, the 3-factor model had slightly lower values of RMSEA and SRMR than the 4-factor model, indicating its superiority as the preferred structure for the SDQ.

### Suggested alternatives

This study showed that a 5-factor structure of the SDQ scale among adolescent Palestinian refugee girls differs from the original 5-factor SDQ scale. Studies in Korea [67] and South Africa [68] also reported the weakness of the 5-factor model among adolescents between 11 and 16 years old. Similar results were reported in studies targeted a wider age range (4–17 years), as in the USA [69], and other studies that targeted younger children (below 10 years), as in Australia [15], the Netherlands [70], and Jordan [71]. In Jordan, for example, the study focused on 4- to 5-year-old children who attend public and private kindergartens. This study demonstrated that the items loaded differently into the 5-factor structure of the teacher version [71]. Furthermore, Dutch and Australian studies reported that the 5-factor structure had a poor to moderate fit, but the 3-latent variables model did not show stronger fit indices [15,70]. On the other hand, a Swedish study used the self-reported SDQ and reported that neither the 5-factor structure nor the 3-factor model provided an accurate fit to data among a sample of 15 and 16-year-olds [72]. However, a modified 5-factor model showed better fit. Unlike our study, the suggested 3-factor SDQ was demonstrated as a better alternative. Several studies support a 3-factor structure, as seen among Greek adolescents (12–17 years), where researchers reported that the 3-factor model was superior to the 4- and 5-factor models, despite some factors having fewer than three items [17]. Another study targeted the same age group, but was conducted in five other European countries, and showed that the 3-factor model fit well in Cyprus, while the 5-factor model fit well in Germany, but neither model fit well in Italy, Sweden, or England [16]. We need to keep in mind that our study differs from the previously reported 3- and 5-factor models in terms of both the distribution of items across factors and the specific items retained.

### Variations in items

Several items central to the original SDQ framework were excluded through the factor analyses. While statistically justified, an additional explanation of the possible reasons for the poor performance of these items might be needed and requires an understanding of the social relations in Palestinian and Arab culture. Palestinians share a collective memory shaped by prolonged suffering, which strengthens their social ties. Palestinian society, as part of the larger Arab community, is collectivist and shows strong ties to religious elements and the extended family [73]. Collectivist societies prefer closely bonded social (often familial) networks and interpersonal harmony [74]. Therefore, adolescents in this study might not feel isolated or lonely even if they do not have a best friend, due to the potential social relations and networks they have with family, cousins, and neighbors. Instead, weak familial relationships might be the main driver of loneliness [74]. This would explain why items like ‘Friend’ and ‘Loner’ did not perform well in the analyses. It can also apply to ‘Adults’ and ‘obedient’ items. Being obedient and respectful toward adults and the elderly, and socializing time with them, is part of the culture and religious values that children and adolescents are raised on [75], rather than an indication of peer problems.

Furthermore, several items were diverted from the factors they should have loaded into, according to the original SDQ, and were instead loaded into other dimensions. The items ‘tantrum’ from conduct problems, ‘loner’ from peer problems, and ‘distractible’ from hyperactivity dimensions loaded onto the emotional problems dimension instead of loading into their designated dimensions. The item ‘loner’, which reflects preferring loneliness, can also fit well within the emotional problems domain. It might not be only literal poor peer communication and interaction; it can also represent an emotional state. Anger and losing temper, while typically considered as conduct issues, often stem from other emotions such as fear, sadness, anxiety, and stress caused by the daily political circumstances. The item ‘distractible’ loading into the emotional problems indicates that adolescents link being distracted and having low concentration with an underlying emotional status. It is reported that distraction can be associated with anxiety and distress. It was also found that distraction can serve as a coping mechanism for overwhelming emotions and traumatic events [76]. A study in Palestine found that higher exposure to political violence among adolescents was associated with developing anxiety, depression, distress, and somatic complaints (internalizing symptoms), as well as aggressive behaviors (externalizing symptoms) [77]. These symptoms are outcomes of structural conditions rather than inherent traits. What can be considered maladaptive in stable conditions can instead be a survival strategy under the chronic violence and structural oppression [76,78].

Popular and attention items from peer problems and hyperactivity domains, respectively, loaded onto the prosocial factor. For our population, popularity might not be just an indicator of the status quo of being liked by the surrounding peers, but it can go deeper to be an expression of being helpful and supportive to others, which eventually can lead to popularity [79]. Popularity can have strong ties with prosocial activity motivations [80]. Some adolescents might be engaged in prosocial behaviors for their self-benefit rather than for the sake of others [80]. Some studies reported a pattern among boys who may engage in prosocial behavior more when they are being observed. This can apply to girls as well, although their prosocial behavior might originate from their nurturing instinct [80]. Another study reported that any type of prosocial behavior will enhance peer relationships [81], possibly leading to higher popularity among peers.

The statement “I finish the work I am doing, my attention is good,” loading to the prosocial can seem counterintuitive. However, it can be linked to prosocial behavior through the concept of self-regulation [81–83]. Such results make sense to appear among the female adolescents in our study. Girls show higher attentional regulation compared to boys [82,83]. According to Eisenberg and colleagues [83], attentional regulation, which is the ability to shift focus and attention, is a foundational skill that is conceptually linked to children’s and adolescents’ prosocial behavior. For example, attention and focus lead to proper task completion, enabling adolescents to help others effectively, in physical tasks, skills development, or even in emotional support [81]. These actions require the ability to stay attentive and committed; qualities that are integral to prosocial interactions.

The last factor consisted of the items bullied, lies, and steals. In the original SDQ, the bullied item belongs to the peer problems domain; however, its loading with the other two items suggests a different latent factor. When the phrasing of the statement ‘Other children or young people pick on me or bully me’ and ‘I am often accused of lying or cheating’ is compared, both are phrased to emphasize actions done by others toward the participants. In conclusion, the shared perspective of being judged or picked on by others can explain why both items might be compatible and co-loaded onto the same latent factor. Although ‘steals’ differs in the way that it emphasizes the respondents’ negative behavior, it can still refer to social and behavioral dysfunction. Victimization by peers (e.g., being bullied) is associated with developing antisocial behaviors such as physical and social aggression and rule-breaking practices [84–86]. Therefore, social dysregulation, where being victimized (Bullied), perceived as dishonest (Lies), and engaging in antisocial behavior (Steals) co-occur.

Finally, while providing cultural explanations, it is important to avoid speculation about the possible reasons that led to some items being eliminated after the EFA or loading differently compared to the original SDQ. Therefore, further research is required to conduct a meticulous exploration of each item’s meaning, message, and relevance through the eyes of Palestinian adolescents themselves, leading to a context-specific SDQ version.

### Convergent, discriminant, and criterion validity

The results of this study do not support the convergent validity of the difficulties SDQ scale. While all the standardized loadings were higher than 0.40 [50], other measures of convergent validity failed to reach the acceptable threshold. The AVE values were all lower than 0.50, indicating that the latent factors account for less than 50% of the variance of their items and indicators [50].

Furthermore, the correlation with the WHO-5 well-being was weak, supporting our conclusion of the poor convergent validity [42,44]. One reason for this may be that both WHO-5 and SDQ measure distinct constructs. SDQ aims to assess emotional and behavioral difficulties, while WHO-5 assesses positive subjective well-being. Also, the WHO-5 assesses well-being over the period of the previous two weeks, while the SDQ captures a broader period. The difference in the recall period for each scale might have influenced the weak correlation. These conceptual and methodological differences may partly explain the weak convergent validity assessed through the WHO-5 scale.

Since convergent validity was not established, it would be meaningless to check the discriminant validity to assess if the ‘difficulties’ can be distinguished from other unrelated constructs (i.e., not correlated with constructs that it should not correlate with theoretically) [50]. This can eventually undermine the construct validity of the proposed three-factor model.

This study provides evidence of the acceptable concurrent validity of the SDQ difficulties. The highest AUC was observed with participants’ overall assessment of difficulties (definite and severe levels). Fair-to-acceptable AUC results were also found with the self-reported psychological or mental health issues diagnosis. In contrast, the AUC results based on life satisfaction and well-being were lower than the acceptable level. The poor AUC for both WHO-5 factors indicates that the SDQ might be more suitable for detecting emotional and behavioral difficulties than for discriminating between adolescents with low well-being or those at risk of depression. The SDQ difficulties might still need refinement, given that the AUC results did not reach the threshold for a good or excellent model [51]. However, interpretation of AUC scores should be approached with caution, given that an agreement on the evaluation criteria is lacking in the literature [51].

The results of the ROC analyses can provide guidance on the possible and most appropriate cut-off points for the difficulties scale in case it is used as a screening tool. For detecting definite and severe overall difficulties, a cut-off point of 8 or 9 emerged with a balanced sensitivity and specificity. A lower cutoff point of 6 or 7 was observed to be optimal for detecting difficulties, regardless of their severity. However, this should be used with caution, given that the sensitivity was higher than the specificity, making it more susceptible to identifying false positives. On the other hand, for identifying psychological or mental health problems, a higher cut-off point was observed (10 or 11). The high specificity compared to the much lower sensitivity suggests that SDQ difficulties might be better at identifying adolescents without psychological or mental health issues than identifying those with such issues. Consequently, while the SDQ may have some use for excluding mental health problems, its relatively lower sensitivity might limit its utility as a tool for identifying adolescents with mental health problems on its own. Finally, the moderate sensitivity and specificity for both WHO-5 factors indicate that using the suggested cut-off points might lead to either missing true positives or misclassifying false positives. These results provide limited support for the criterion validity of the SDQ based on the WHO-5 as an external criterion.

In conclusion, our results imply that utilizing this scale in clinical settings should be done with caution, despite its better structure compared to the original SDQ. Although the SDQ is being used as a screening tool in clinical settings to assess adolescents’ mental health and provide information for health care professionals to diagnose psychopathology or disorders [3], there are calls for practitioners to cease using the current form of SDQ as a screening tool [3]. A study targeted Aboriginal people in Australia recommended avoiding using the summation of subscale scores for clinical screening [15]. One study in England called for caution in using SDQ to screen for ADHD among children [52]. Therefore, exploring SDQ’s psychometric properties in both clinical and community settings is unavoidable to examine its suitability for clinical diagnosis or its usefulness as a screening instrument [87].

### Reliability

To measure the internal consistency of the suggested alternative, we conducted several tests. These tests indicated that reliability was strongest for Factor 1 (emotional distress), indicating consistent measurement, given that α, ω, and λ2 were higher than 0.7 [42,44,88]. The average inter-item correlations fell within the recommended range, which is between 0.15 and 0.5 [62], and the item-to-total correlations are ≥ 0.40 (except for two items that were very close to this threshold), add more support to the good internal consistency status of Factor 1.

For factors 2 and 3, the judgment was tricky. Both factors had α, ω, and λ2 values lower than 0.7, but they were 0.60 or slightly lower. Although the most commonly used threshold is 0.70 for acceptable internal consistency [42,44,88,89], it was also reported that Cronbach’s alpha values ≥0.60 can be acceptable [90]. Based on this, from a stricter perspective, their internal consistency can be judged as questionable or poor. However, both factors can be considered to have marginal but acceptable internal consistency if we include the results of the average inter-item and the item-to-total correlations for both factors. Both showed good average inter-item correlations and showed marginal to acceptable item-to-total correlations. Overall, while Factor 1 (emotional distress) is robust, Factor 2 (prosocial) and Factor 3 (conduct) subscales and items may require refinement to achieve stronger reliability. Improving the reliability of the scale would potentially improve the scale’s potential for use, including in clinical settings for screening and/or diagnosis. Low reliability coefficients might lead to measurement errors and contribute to the misclassification of adolescents’ true status. Therefore, clinical decisions based on scores calculated from non-reliable scales, especially when dealing with adolescents, should be strongly discouraged [3].

### Strengths and limitations

This study is not without limitations. First, the sample included only adolescent girls, which limits the generalizability of this study’s results to the broader Palestinian adolescent refugee population. Acknowledging the possible nuanced differences between boys and girls in their reaction to the stressors necessitates having a more rigorous validation process and development of items that account for gender-specific experiences. Therefore, the applicability of this scale among boys cannot be assumed and still needs to be established. However, future research to test this scale’s validity among males, a mixed sample, and among younger adolescents is necessary. The final 3-factor alternative is comprised of 15 items, meaning 10 items were dropped from the original SDQ scale. This would minimize the ability to capture many of the constructs that were meant to be measured. However, dropping those items was based on a meticulous statistical process to ensure that the models had a better fit, which provides evidence of improved construct validity. Another possible limitation is that some items are loaded into factors other than the ones they should be loaded onto. These loadings might look counterintuitive at first glance, but this can be due to the double meaning that some of these items can carry. We also provided justification and explanation supported by previous research and the context of those adolescents to show that the item clusters formed make sense. However, further research is still needed to ensure that the suggested alternative is robust enough to work with other samples. Finally, the variables used to evaluate convergent, discriminant, and criterion validity may not be perfect measures. For example, the self-reported diagnosis of a psychological or mental illness question might give an underestimation of mental illness levels because many people tend not to seek professional mental health treatment or not to report their actual condition, and this might have an impact on the results. The large sample size is a strength of this study, which improves the generalizability of the results among adolescent girls in Palestine refugee camps and increases the reliability of the results.

## Conclusion

The original SDQ structures’ validity and reliability were not supported to be used among adolescent Palestinian refugee girls in the West Bank and Jordan. Instead, the 3-factor and 15-item alternative might be a better alternative, given its superior fit indices and better construct validity. However, this alternative SDQ is still far from being described as a good substitute for the original one, given that it has poor convergent validity, fair to acceptable concurrent validity, and barely acceptable reliability. This study also implies how contexts, culture, and the surrounding environment influence the way that adolescents in refugee settings understand, interpret, and respond to SDQ items. This was evident through the variations in item loadings compared to the original SDQ structure, emphasizing that applying SDQ in its current form is challenging. While the suggested 3-factor alternative provides a more fitting model for assessing difficulties, specifically emotional ones, among adolescent refugees, utilizing this scale should be done with caution, especially in clinical settings, whether for diagnosis or screening. Our findings highlight the importance of validating mental health screening instruments before their implementation in humanitarian settings. These findings underscore the need for psychometrically robust and contextually relevant screening tools to support accurate identification of adolescents who may benefit from further mental health assessment and appropriate referral.

Therefore, this scale requires further refinements and adjustments. For instance, incorporating items that reflect the context refugees live in, like war-related trauma or items related to discrimination, would make the SDQ is more capable of detecting the full range of mental health difficulties prevalent in vulnerable populations such as refugees. To enhance SDQ’s validity, reliability, and relevance for this population, future research should prioritize cognitive interviews to have a clear understanding of how each item is interpreted and comprehended among this population. This would ensure that problematic items are either revised, removed, or replaced. Furthermore, future research should conduct further validation of the SDQ in other war zones or conflict areas to ensure that the scale is valid and reliable. This validation process should be conducted in both community and clinical settings.

## Supporting information

S1 Appendix Sample size calculation and determination. (DOCX)

S2 Checklist Inclusivity checklist. (PDF)

S3 Checklist Human participation checklist. (PDF)
