# Supplementary material for: Exploring psychometric properties of the Strengths and Difficulties Questionnaire (SDQ) among adolescent Palestinian refugee girls in Palestine and Jordan
Source: PLoS One. 2026 Sep 25;21(9):e0349999. doi: 10.1371/journal.pone.0349999 (PMC13614631; doi:10.1371/journal.pone.0349999)
Supplement: Supplementary file 1 [file pone.0349999.s002.pdf]

Inclusivity in global research

PLOS' policy on inclusivity in global research aims to improve transparency in the reporting of research performed outside of researchers' own country or community and ensures that PLOS publications reporting global research adhere to high standards for research ethics and authorship. Authors of relevant research articles may be asked to complete the questionnaire below, which outlines ethical, cultural, and scientific considerations specific to inclusivity in global research. This questionnaire may be requested when researchers have travelled to a different country to conduct research, if research uses samples collected in another country, research with Indigenous populations or their lands, or if research is on cultural artefacts. Researchers travelling to another country solely to use laboratory equipment will not normally be required to complete the questionnaire. However, the questionnaire can be requested at the journal's discretion for any submission – if you have been requested to complete this questionnaire by the PLOS journal you submitted to, please do so.

Please complete the questionnaire below and include this as a Supporting Information file with your manuscript. Note that if your paper is accepted for publication, this checklist will be published with your article in the supporting information files. Please ensure that you reference the checklist in the main body of your manuscript. We suggest adding a subsection 'Inclusivity in global research' to your Methods section and adding the following sentence: "Additional information regarding the ethical, cultural, and scientific considerations specific to inclusivity in global research is included in the Supporting Information (SX Checklist)"

The questions have been designed to be applicable to a wide range of study types, and there are subsections for both human subjects research and non-human subjects research. If any of the questions are not relevant to your research please mark them as "N/A" as appropriate.

Ethical considerations, permits and authorship

This section is applicable to all research types.

Provide details as to who granted permissions and/or consent for the study to take place in the Methods section of your manuscript. This should include the names of **all** ethics boards, governmental organizations, community leaders or other bodies that provided approval for the study. If individuals provided approval refer to these people by their role or title but do not list their name(s).

Reported on page number: 13

If there were any deviations from the study protocol after approval was obtained please provide details of these changes in the Methods section of your manuscript.

Reported on page number: N/A

Did this study involve local collaborators that are residents of the country where the research was conducted or members of the community studied? If you do not have any authors from said communities, please provide an explanation for this below.

Yes. The study was led by local researchers with extensive experience working with Palestinian refugee communities. Two of the authors (Rula Ghandour and Weeam Hammoudeh), both based at Birzeit University in Palestine, were directly involved in the design and implementation of the study, including oversight of the fieldwork and participation in data collection. Data collection was carried out by trained female fieldworkers who were either residents of the refugee camps or highly familiar with the local context. This manuscript reports a psychometric validation study, and authorship reflects substantial intellectual contributions to the study design, implementation, analysis, interpretation of the findings, and manuscript preparation, in accordance with standard authorship criteria.

Everyone listed as an author should meet PLOS' criteria for authorship and all individuals who meet these criteria should be included in the author byline, rather than the acknowledgements. For further information please see the journal's Authorship Policy.

Human subjects research (e.g. health research, medical research, cross-cultural psychology)

Did you obtain written informed consent from a representative of the local community or region before the research took place? How did you establish who speaks for the community? Details of written informed consent obtained from study participants should be reported separately in the Methods section of your manuscript.

Yes. Before data collection began, approvals and permissions to conduct the study were obtained from the relevant institutional and community representatives in both study settings. In Palestine, approval was obtained from UNRWA, the Executive Office for Palestinian Refugees and the Popular Committees in the West Bank, and the local Popular Committees within the refugee camps. In Jordan, permission was obtained from UNRWA and the Department of Palestinian Affairs (DPA), which is the governmental body responsible for the administration of Palestinian refugee camps.

These institutions and committees were identified as the appropriate representatives because they are the officially recognized authorities responsible for the governance, administration, and coordination of services within the refugee camps, and they facilitate access to the communities. Their approval was obtained before approaching households for recruitment. Written informed consent from participants and their guardians was obtained separately, as described in the Methods section of the manuscript.

How did members of the local community provide input on the aims of the research investigation, its methodology, and its anticipated outcome(s)?

Community input was incorporated into the research throughout the study development process. Before the quantitative phase, an exploratory qualitative phase was conducted, including in-depth interviews with adolescent girls, mothers, community representatives, service providers, and other key stakeholders living and working in the Palestinian refugee camps. These discussions helped identify the priority health concerns, contextual factors, and issues that were most relevant to adolescent girls, and informed the development of the quantitative study objectives and data-collection tools.

Before implementation, a launching meeting was held with UNRWA representatives to discuss the study objectives, methodology, implementation plan, and expected outcomes. Additional meetings were conducted with the relevant authorities and community representatives, including the Popular Committees in the West Bank and the Department of Palestinian Affairs in Jordan, to discuss the study procedures, ensure that the research addressed local priorities, and facilitate its implementation within the refugee camps. Feedback received during these consultations was incorporated into the final study design and implementation plan.

When engaging with the local community, how did you ensure that the informed consent documents and other materials could be understood by local stakeholders?

All participant-facing materials, including the participant information sheets, informed consent forms, assent forms, and questionnaires, were prepared in Arabic, the native language of the study participants and community stakeholders. The materials were developed using language that was appropriate for the target population and were reviewed and pilot-tested to ensure clarity, contextual relevent, and ease of understanding.

Data collection was conducted by trained female fieldworkers who were either residents of the refugee camps or highly familiar with the local context. Before obtaining written informed consent (and assent for minors), the fieldworkers explained the study objectives, procedures, potential risks and benefits, confidentiality, and participants' rights using clear and culturally appropriate language. Participants and parents or legal guardians were given sufficient time to ask questions and were informed that participation was voluntary and that they could decline or withdraw at any time without any consequences. This approach helped ensure that informed consent was based on a clear understanding of the study.

Will the findings of the research be made available in an understandable format to stakeholders in the community where the study was conducted (e.g. via a presentation, summary report, copies of publications, etc.)? Please provide details of how this will be achieved.

Yes. Given the nature of this study, the primary audience for the findings includes researchers, clinicians, and public health professionals who may use the validated tool for screening and research. The findings will therefore be disseminated through peer-reviewed publications, scientific conferences, and professional networks to facilitate the uptake and appropriate use of the instrument.

In addition, an Arabic summary of the main findings will be shared with UNRWA and the collaborating institutions involved in the study. This summary will present the key results in accessible language and highlight the implications of the validated tool for screening and research among adolescent girls in Palestinian refugee camps.

Non-human subjects research using specimens/ animals collected as part of the study, or those housed in archival collections. Examples include archaeology, paleontology, botany and zoology.

Did the permission you obtained from a local authority to perform the study include an agreement on access to outputs and benefit sharing? This may include procedures to enable fair distribution of the benefits and resources arising from the research performed. Please include any details of Prior Informed Consent and Benefit Sharing Agreements obtained. These may be required by field-specific regulations, for example the Convention on Biological Diversity (CBD) and the associated Nagoya Protocol.

N/A

If the material used in your study was imported, please A) provide the year it was imported and B) indicate whether permits were obtained to import/export the materials used, C) provide details of any permits obtained. If this information is not available, please indicate this.

N/A

If you used archival specimens, please state how the material used in your study was acquired by the institute it is held in and provide details of any permits obtained for the original excavations/ sample collection. If this information is not available, please indicate this.

N/A

How was the potential cultural significance of the materials collected in your study to local communities considered in your research design? Were Indigenous peoples and/or local researchers and institutions involved with archaeological excavations / collection of specimens? If so, please provide a description of their involvement.

N/A

If your manuscript includes photographs of human remains please indicate whether authors obtained permission from descendants or affiliated cultural communities to do so.

N/A
