# Supplementary material for: Exploring psychometric properties of the Strengths and Difficulties Questionnaire (SDQ) among adolescent Palestinian refugee girls in Palestine and Jordan
Source: PLoS One. 2026 Sep 25;21(9):e0349999. doi: 10.1371/journal.pone.0349999 (PMC13614631; doi:10.1371/journal.pone.0349999)
Supplement: Supplementary file 3 [file pone.0349999.s001.docx]

**Supplement 1: Sample size calculation and determination**

The study employed a **stratified random sampling** approach with **proportional allocation**. Although a simple random sampling (SRS) design was the baseline for the initial calculation, a **Design Effect (deff) of 2** was applied to the final requirement.

This adjustment was integrated to account for the potential homogeneity within the unique environment of refugee camps. Because participants within the same camp often share similar socio-economic conditions and health access, they may exhibit higher correlation than a truly independent national sample. Using a deff of 2 ensures the study is sufficiently powered to overcome this "design inefficiency" and provides a more conservative, robust estimate of anemia prevalence (r).

The formula used for the calculation was:

$$n=\frac{{1.96}^{2}*r\left( 1-r \right)*deff}{({0.15r)}^{2}}$$

Where

- r: is the prevalence of anemia, the lowest expected indicator in the bigger project from which the data was obtained (See methods section)
- deff: The design effect constant (2) used to ensure statistical power despite non-independence of observations within strata
- 0.15: margin of error tolerated at 95% confidence interval

The sample was calculated independently for each region to account for geographic differences in anemia levels:

- **West Bank of Palestine:** n = 1,456
- **Jordan:** n = 972
- **Total Calculated Target:** **2,428**

**Participant Enrollment and Final Cohort**

Although the statistical requirement was 2,428, field teams successfully engaged **2,949** girls. Following a rigorous screening process—which included verifying age (15–18 years), confirming Palestinian refugee status, and ensuring questionnaire completion—a final analytical sample of **2,725** participants was established.

**Recruitment Protocol**

Enrollment followed a systematic, tiered consent process:

1. **Household Entry:** Researchers initially requested participation from female household heads to complete a general household roster, regardless of the presence of adolescents.
2. **Guardian Permission:** In households with eligible girls aged 15–18, permission was sought from the household head to interview all resident adolescent girls.
3. **Individual Assent:** After securing household-level consent, researchers obtained direct, voluntary approval from the girls themselves before beginning the interview process.
