# Supplementary material for: Exploring psychometric properties of the Strengths and Difficulties Questionnaire (SDQ) among adolescent Palestinian refugee girls in Palestine and Jordan
Source: PLoS One. 2026 Sep 25;21(9):e0349999. doi: 10.1371/journal.pone.0349999 (PMC13614631; doi:10.1371/journal.pone.0349999)
Supplement: Peer review history

# Peer review: Decision Letter 0

**DOI:** 10.1371/journal.pone.0349999.r001

**Authors:** Harikrishnan U

12 Jun 2026

PONE-D-26-18251

Exploring psychometric properties of the Strengths and Difficulties (SDQ) among Adolescent Palestinian refugee girls in Palestine and Jordan

PLOS One

Dear Dr. Ghandour,

Thank you for submitting your manuscript to PLOS ONE. After careful consideration, we feel that it has merit but does not fully meet PLOS ONE’s publication criteria as it currently stands. Therefore, we invite you to submit a revised version of the manuscript that addresses the points raised during the review process.

==============================

Dear Author,

I appreciate the effort you put into the manuscript. The reviewers have suggested that revisions are needed, and I agree with their assessment. Please revise the manuscript according to the journal’s format and cross-check it with the author guidelines before resubmission.

==============================

Please submit your revised manuscript by Jul 26 2026 11:59PM. If you will need more time than this to complete your revisions, please reply to this message or contact the journal office at plosone@plos.org. When you’re ready to submit your revision, log on to https://www.editorialmanager.com/pone/ and select the 'Submissions Needing Revision' folder to locate your manuscript file.

Please include the following items when submitting your revised manuscript:

- A letter that responds to each point raised by the academic editor and reviewer(s). You should upload this letter as a separate file labeled 'Response to Reviewers'.
- A marked-up copy of your manuscript that highlights changes made to the original version. You should upload this as a separate file labeled 'Revised Manuscript with Track Changes'.
- An unmarked version of your revised paper without tracked changes. You should upload this as a separate file labeled 'Manuscript'.

If you would like to make changes to your financial disclosure, please include your updated statement in your cover letter. Guidelines for resubmitting your figure files are available below the reviewer comments at the end of this letter.

If applicable, we recommend that you deposit your laboratory protocols in protocols.io to enhance the reproducibility of your results. Protocols.io assigns your protocol its own identifier (DOI) so that it can be cited independently in the future. For instructions see: https://journals.plos.org/plosone/s/submission-guidelines#loc-laboratory-protocols. Additionally, PLOS ONE offers an option for publishing peer-reviewed Lab Protocol articles, which describe protocols hosted on protocols.io. Read more information on sharing protocols at https://plos.org/protocols?utm_medium=editorial-email&utm_source=authorletters&utm_campaign=protocols.

As the corresponding author, your ORCID iD is verified in the submission system and will appear in the published article. PLOS supports the use of ORCID, and we encourage all coauthors to register for an ORCID iD and use it as well. Please encourage your coauthors to verify their ORCID iD within the submission system before final acceptance, as unverified ORCID iDs will not appear in the published article. Only the individual author can complete the verification step; PLOS staff cannot verify ORCID iDs on behalf of authors.

We look forward to receiving your revised manuscript.

Kind regards,

Harikrishnan U, Ph.D

Academic Editor

PLOS One

Journal Requirements:

When submitting your revision, we need you to address these additional requirements.

1. Please ensure that your manuscript meets PLOS ONE's style requirements, including those for file naming. The PLOS ONE style templates can be found at

https://journals.plos.org/plosone/s/file?id=wjVg/PLOSOne_formatting_sample_main_body.pdf and

https://journals.plos.org/plosone/s/file?id=ba62/PLOSOne_formatting_sample_title_authors_affiliations.pdf

2. Please provide additional information regarding the considerations  made for the refugees included in this study. For instance, please discuss whether participants were able to opt out of the study and whether individuals who did not participate receive the same treatment offered to participants

3. Please include a complete copy of PLOS’ questionnaire on inclusivity in global research in your revised manuscript. Our policy for research in this area aims to improve transparency in the reporting of research performed outside of researchers’ own country or community. The policy applies to researchers who have travelled to a different country to conduct research, research with Indigenous populations or their lands, and research on cultural artefacts. The questionnaire can also be requested at the journal’s discretion for any other submissions, even if these conditions are not met. Please find more information on the policy and a link to download a blank copy of the questionnaire here: https://journals.plos.org/plosone/s/best-practices-in-research-reporting. Please upload a completed version of your questionnaire as Supporting Information when you resubmit your manuscript.”

3. Thank you for stating the following financial disclosure:

“This work was supported by the International Development Research Center (IDRC) in Ottawa, Canada. The views expressed here do not necessarily represent those of the IDRC or its Board of Governance.

Project ID  108672 (2018-2023)”

Please state what role the funders took in the study.  If the funders had no role, please state: "The funders had no role in study design, data collection and analysis, decision to publish, or preparation of the manuscript."

If this statement is not correct you must amend it as needed.

Please include this amended Role of Funder statement in your cover letter; we will change the online submission form on your behalf.

4. We note that you have indicated that there are restrictions to data sharing for this study. PLOS only allows data to be available upon request if there are legal or ethical restrictions on sharing data publicly. For more information on unacceptable data access restrictions, please see http://journals.plos.org/plosone/s/data-availability#loc-unacceptable-data-access-restrictions.

Before we proceed with your manuscript, please address the following prompts:

a) If there are ethical or legal restrictions on sharing a de-identified data set, please explain them in detail (e.g., data contain potentially identifying or sensitive patient information, data are owned by a third-party organization, etc.) and who has imposed them (e.g., a Research Ethics Committee or Institutional Review Board, etc.). Please also provide contact information for a data access committee, ethics committee, or other institutional body to which data requests may be sent.

b) If there are no restrictions, please upload the minimal anonymized data set necessary to replicate your study findings to a stable, public repository and provide us with the relevant URLs, DOIs, or accession numbers. For a list of recommended repositories, please see

https://journals.plos.org/plosone/s/recommended-repositories. You also have the option of uploading the data as Supporting Information files, but we would recommend depositing data directly to a data repository if possible.

We will update your Data Availability statement on your behalf to reflect the information you provide.

5. Please include captions for your Supporting Information files at the end of your manuscript, and update any in-text citations to match accordingly. Please see our Supporting Information guidelines for more information: http://journals.plos.org/plosone/s/supporting-information.

6. If the reviewer comments include a recommendation to cite specific previously published works, please review and evaluate these publications to determine whether they are relevant and should be cited. There is no requirement to cite these works unless the editor has indicated otherwise.

7. Please review your reference list to ensure that it is complete and correct. If you have cited papers that have been retracted, please include the rationale for doing so in the manuscript text, or remove these references and replace them with relevant current references. Any changes to the reference list should be mentioned in the rebuttal letter that accompanies your revised manuscript. If you need to cite a retracted article, indicate the article’s retracted status in the References list and also include a citation and full reference for the retraction notice.

[Note: HTML markup is below. Please do not edit.]

Reviewers' comments:

Reviewer's Responses to Questions

**Comments to the Author**

1. Is the manuscript technically sound, and do the data support the conclusions?

Reviewer #1: Yes

Reviewer #2: Yes

Reviewer #3: Yes

**********

2. Has the statistical analysis been performed appropriately and rigorously?

Reviewer #1: Yes

Reviewer #2: Yes

Reviewer #3: Yes

**********

3. Have the authors made all data underlying the findings in their manuscript fully available?

The PLOS Data policy

Reviewer #1: Yes

Reviewer #2: Yes

Reviewer #3: No

**********

4. Is the manuscript presented in an intelligible fashion and written in standard English?

Reviewer #1: Yes

Reviewer #2: Yes

Reviewer #3: Yes

**********

Reviewer #1: Reviewer Comments (Approx. 500 Words)

Manuscript Title: Exploring Psychometric Properties of the Strengths and Difficulties Questionnaire (SDQ) among Adolescent Palestinian Refugee Girls in Palestine and Jordan

This manuscript addresses an important and underexplored area in adolescent mental health assessment among refugee populations. The study evaluates the psychometric properties of the Strengths and Difficulties Questionnaire (SDQ) among a large sample of Palestinian refugee adolescent girls residing in refugee camps in the West Bank and Jordan. The sample size is substantial (n = 2,725), providing adequate statistical power for exploratory and confirmatory factor analyses and strengthening the credibility of the findings.

A major strength of the study is its comprehensive psychometric evaluation, including construct validity through EFA and CFA, convergent validity, criterion validity using ROC analyses, and multiple reliability indicators (Cronbach’s alpha, McDonald’s omega, Guttman’s lambda-2). The authors demonstrate that the original five-factor SDQ structure shows poor fit in this population, while an alternative three-factor, 15-item model exhibits substantially improved fit indices. These findings contribute meaningfully to the literature on cross-cultural validation of mental health screening tools.

However, several issues require attention before publication.

First, the manuscript occasionally overstates the conclusion that the original SDQ is “neither valid nor reliable.” While the CFA results indeed indicate poor structural fit, some reliability indicators remain acceptable, particularly for the overall difficulties score. Therefore, the wording should be moderated to reflect that the original factor structure was not supported rather than implying complete invalidity of the instrument.

Second, the study sample includes only adolescent girls aged 15–18 years. This limitation significantly affects the generalizability of the findings. The manuscript should emphasize more clearly that conclusions cannot be extended to boys, younger adolescents, or non-refugee Palestinian populations.

Third, the rationale for item removal could be strengthened. Several items central to the original SDQ framework (e.g., Obedient, Friend, Fights, Reflective) were excluded due to low factor loadings. While statistically justified, additional theoretical and cultural explanations would help readers understand why these items performed poorly within refugee camp contexts.

Fourth, convergent validity findings are weak. The reported correlation between SDQ difficulties and WHO-5 well-being was only r = –0.245, and AVE values remained below recommended thresholds. This raises concerns regarding the construct validity of the proposed three-factor model. The discussion should elaborate more critically on the implications of these findings and whether the alternative model is suitable for practical screening purposes.

Additionally, the use of self-reported mental health diagnosis and self-perceived difficulties as criterion measures may introduce reporting bias. Inclusion of clinician-administered diagnostic assessments would have provided a stronger external validation criterion.

Finally, the manuscript would benefit from language editing. There are several grammatical inconsistencies, repetition, and minor typographical errors throughout the introduction and discussion sections. Careful proofreading would improve readability and overall presentation.

Recommendation: Minor Revision

The study addresses an important gap and offers valuable evidence regarding SDQ use among Palestinian refugee adolescents. However, concerns regarding validity interpretation, theoretical justification of item reduction, generalizability, and presentation should be addressed before the manuscript is suitable for publication.

Reviewer #2: Importance and Justification

There should be a stronger justification for concentrating only on teenage girls. The consequences of leaving out males and the ensuing restrictions on generalizability should be covered by the investigators.

Clarity of Methodology

The research design, sampling methods, participant recruitment processes, and inclusion/exclusion criteria require further clarification.

A more thorough explanation of the item elimination procedure in the alternative models should be provided, together with theoretical support and statistical standards

Evaluation of Validity

Further discussion is necessary given the WHO-5 Well-Being Index's limited convergent validity. The authors should investigate potential methodological, cultural, or contextual factors underlying this result.

Interpretation would be improved by more discussion of the reported AUC values' practical significance and criterion validity.

Issues with Reliability

The three-factor model showed better fit indices, but its dependability was only moderate. The authors should discuss the suitability of the suggested model for clinical or screening applications.

Minor suggestions

1. Consider creating a summary table with the final three-factor model's 15 items that were kept.

2. The study's application might be increased with a brief explanation of the practical implications for mental health screening programs in refugee situations.

Reviewer #3: (1) It's a good attempt to highlight the SDQ model with its pros and cons.

(2) This study has used data from a wider study on the targeted population. It needs to present the basic socio-economic profile of the respondents, i.e., adolescents and their families, for better understanding by readers.

(3) Reviews have been done appropriately to supplement a sufficient argument to the core argument.

(4) However, study objectives and core research questions have not been mentioned properly.

(5) In Methodology, it is written that the study has been conducted in three phases – Qualitative, Quantitative and Intervention Phases; further, it mentioned the "Structures Survey Tools" that were used to collect data. It's better to mention "semi-structured tools", or it may be justified by the author.

(6) The analysis part is well explained, giving priority to validating each point of the SDQ, whether they are worth probing or not, thus drawing the acceptance or modification of the model (ADQ) itself.

(7) At the same time, can it be more reader-friendly? Extensive analysis creates more critical understanding for the readers.

(8) Limitations have been well placed. The SDQ model has its own limitations, and could be modified or replaced by a better model over time and tested in different settings.

**********

what does this mean?). If published, this will include your full peer review and any attached files.

If you choose “no”, your identity will remain anonymous but your review may still be made public.

**Do you want your identity to be public for this peer review?**  For information about this choice, including consent withdrawal, please see our Privacy Policy

Reviewer #1: No

Reviewer #2: **Yes:**  Dr Rajeev MM Nambiar

Reviewer #3: No

**********

[NOTE: If reviewer comments were submitted as an attachment file, they will be attached to this email and accessible via the submission site. Please log into your account, locate the manuscript record, and check for the action link "View Attachments". If this link does not appear, there are no attachment files.]

To ensure your figures meet our technical requirements, please review our figure guidelines: https://journals.plos.org/plosone/s/figures

You may also use PLOS’s free figure tool, NAAS, to help you prepare publication quality figures: https://journals.plos.org/plosone/s/figures#loc-tools-for-figure-preparation.

NAAS will assess whether your figures meet our technical requirements by comparing each figure against our figure specifications.

# Author comment: Author response to Decision Letter 1

**DOI:** 10.1371/journal.pone.0349999.r002

8 Aug 2026

Response to review comments

Editor:

When submitting your revision, we need you to address these additional requirements.

1. Please ensure that your manuscript meets PLOS ONE's style requirements, including those for file naming. The PLOS ONE style templates can be found at

https://journals.plos.org/plosone/s/file?id=wjVg/PLOSOne_formatting_sample_main_body.pdf and

https://journals.plos.org/plosone/s/file?id=ba62/PLOSOne_formatting_sample_title_authors_affiliations.pdf

Thank you. The manuscript has been updated to ensure adherence to these guidelines.

2. Please provide additional information regarding the considerations made for the refugees included in this study. For instance, please discuss whether participants were able to opt out of the study and whether individuals who did not participate receive the same treatment offered to participants

Thank you, we have added further clarification in the text. Page 8 line 207 we added:

All individuals approached to participate in the study were informed that participation was entirely voluntary and that they could withdraw from the study at any time without any consequences. They were also assured that their decision to participate, decline participation, or withdraw would not affect their access to any services. Consent was obtained in two stages. First, informed consent was obtained from the mother or another adult guardian in the household. Second, informed assent/consent was obtained from the adolescent girl herself. At both stages, participants were informed that they could decline participation altogether or choose not to answer any question they did not wish to answer.

3. Please include a complete copy of PLOS’ questionnaire on inclusivity in global research in your revised manuscript. Our policy for research in this area aims to improve transparency in the reporting of research performed outside of researchers’ own country or community. The policy applies to researchers who have travelled to a different country to conduct research, research with Indigenous populations or their lands, and research on cultural artefacts. The questionnaire can also be requested at the journal’s discretion for any other submissions, even if these conditions are not met. Please find more information on the policy and a link to download a blank copy of the questionnaire here: https://journals.plos.org/plosone/s/best-practices-in-research-reporting. Please upload a completed version of your questionnaire as Supporting Information when you resubmit your manuscript.”

Thank you. The section has been added to the methods section, page 13, Line 344. And also the questionnaire has been added as a supplement (S2)

3. Thank you for stating the following financial disclosure:

“This work was supported by the International Development Research Center (IDRC) in Ottawa, Canada. The views expressed here do not necessarily represent those of the IDRC or its Board of Governance. Project ID 108672 (2018-2023)”

Please state what role the funders took in the study. If the funders had no role, please state: "The funders had no role in study design, data collection and analysis, decision to publish, or preparation of the manuscript."

If this statement is not correct, you must amend it as needed.

Please include this amended Role of Funder statement in your cover letter; we will change the online submission form on your behalf.

Thank you. The statement you suggested has been added to the funding information page 30.

4. We note that you have indicated that there are restrictions to data sharing for this study. PLOS only allows data to be available upon request if there are legal or ethical restrictions on sharing data publicly. For more information on unacceptable data access restrictions, please see http://journals.plos.org/plosone/s/data-availability#loc-unacceptable-data-access-restrictions.

Before we proceed with your manuscript, please address the following prompts:

a) If there are ethical or legal restrictions on sharing a de-identified data set, please explain them in detail (e.g., data contain potentially identifying or sensitive patient information, data are owned by a third-party organization, etc.) and who has imposed them (e.g., a Research Ethics Committee or Institutional Review Board, etc.). Please also provide contact information for a data access committee, ethics committee, or other institutional body to which data requests may be sent.

There are ethical restrictions on sharing the de-identified dataset imposed by the Research Ethics Committee (REC) at Birzeit University as part of the study approval. Although the dataset has been de-identified, it contains sensitive information collected from adolescent girls living in Palestinian refugee camps. Given the vulnerability of the study population and the politically sensitive context in which the data were collected, unrestricted public sharing could increase the risk of unintended disclosure or misuse of the data.

The dataset is therefore not publicly available. Researchers who wish to access the data for scientifically and ethically appropriate purposes may submit a request on a case-by-case basis to the principal investigators, Dr. Rula Ghandour or Dr. Weeam Hammoudeh. Requests will be reviewed in accordance with the conditions of the ethical approval granted by the Research Ethics Committee at Birzeit University and any applicable institutional requirements.

b) If there are no restrictions, please upload the minimal anonymized data set necessary to replicate your study findings to a stable, public repository and provide us with the relevant URLs, DOIs, or accession numbers. For a list of recommended repositories, please see

https://journals.plos.org/plosone/s/recommended-repositories. You also have the option of uploading the data as Supporting Information files, but we would recommend depositing data directly to a data repository if possible.

We will update your Data Availability statement on your behalf to reflect the information you provide.

5. Please include captions for your Supporting Information files at the end of your manuscript, and update any in-text citations to match accordingly. Please see our Supporting Information guidelines for more information: http://journals.plos.org/plosone/s/supporting-information.

We changed the in-text citation from Supplementary 1 to S1 Appendix, and we added the caption at the end of the manuscripts requested. We did the same the global research inclusivity supplement S2.

6. If the reviewer comments include a recommendation to cite specific previously published works, please review and evaluate these publications to determine whether they are relevant and should be cited. There is no requirement to cite these works unless the editor has indicated otherwise.

This was not applicable. However, we added several citations for the newly added text based on reviewers’ comments.

7. Please review your reference list to ensure that it is complete and correct. If you have cited papers that have been retracted, please include the rationale for doing so in the manuscript text, or remove these references and replace them with relevant current references. Any changes to the reference list should be mentioned in the rebuttal letter that accompanies your revised manuscript. If you need to cite a retracted article, indicate the article’s retracted status in the References list and also include a citation and full reference for the retraction notice.

The reference list was revised, and no retracted papers were cited. We cited additional papers while addressing reviewers' comments, and we replaced some papers with others. For example, we replaced “Ahmad N, Alias FA, Hamat M, Mohamed SA. Reliability analysis: application of cronbach’s alpha in research instruments. Pioneer Futur Delving Into E‐Learning’s Landsc. 2024;114–9” with “Taber KS. The Use of Cronbach’s Alpha When Developing and Reporting Research Instruments in Science Education. Res Sci Educ [Internet]. 2018;48(6):1273–96. Available from: https://doi.org/10.1007/s11165-016-9602-2”

Review Comments to the Author

Please use the space provided to explain your answers to the questions above. You may also include additional comments for the author, including concerns about dual publication, research ethics, or publication ethics. (Please upload your review as an attachment if it exceeds 20,000 characters)

Reviewer #1:

Manuscript Title: Exploring Psychometric Properties of the Strengths and Difficulties Questionnaire (SDQ) among Adolescent Palestinian Refugee Girls in Palestine and Jordan

This manuscript addresses an important and underexplored area in adolescent mental health assessment among refugee populations. The study evaluates the psychometric properties of the Strengths and Difficulties Questionnaire (SDQ) among a large sample of Palestinian refugee adolescent girls residing in refugee camps in the West Bank and Jordan. The sample size is substantial (n = 2,725), providing adequate statistical power for exploratory and confirmatory factor analyses and strengthening the credibility of the findings.

A major strength of the study is its comprehensive psychometric evaluation, including construct validity through EFA and CFA, convergent validity, criterion validity using ROC analyses, and multiple reliability indicators (Cronbach’s alpha, McDonald’s omega, Guttman’s lambda-2). The authors demonstrate that the original five-factor SDQ structure shows poor fit in this population, while an alternative three-factor, 15-item model exhibits substantially improved fit indices. These findings contribute meaningfully to the literature on cross-cultural validation of mental health screening tools.

Thank you for the efforts made by the reviewer to provide us with valuable comments and feedback that helped us to improve the quality of the manuscript.

However, several issues require attention before publication.

1. First, the manuscript occasionally overstates the conclusion that the original SDQ is “neither valid nor reliable.” While the CFA results indeed indicate poor structural fit, some reliability indicators remain acceptable, particularly for the overall difficulties score. Therefore, the wording should be moderated to reflect that the original factor structure was not supported, rather than implying complete invalidity of the instrument.

Thank you for pointing this out. Our intention was neither to generalize this statement to all populations nor to imply that the scale was entirely useless. Rather, we wanted to show that its structure did not do well in terms of validity and reliability when tested among Palestinian refugees in the West Bank and Jordan. However, to avoid any implication, we revised the statement as suggested:

“The original SDQ structures’ validity and reliability were not supported to be used among Palestinian adolescent refugee girls in the West Bank and Jordan.”

2. Second, the study sample includes only adolescent girls aged 15–18 years. This limitation significantly affects the generalizability of the findings. The manuscript should emphasize more clearly that conclusions cannot be extended to boys, younger adolescents, or non-refugee Palestinian populations.

We addressed the limitation of generalizability in the ‘Strengths and limitations’ section. Also, in the conclusion, we already have the statement ‘This study also implies how contexts, culture, and the surrounding environment influence the way that adolescents in refugee settings understand, interpret, and respond to SDQ items.’

We mistakenly had “Therefore, we can say that it might also apply to boys” which should be “Therefore, we cannot say that it might also apply to boys”. Therefore, we revised this part as follows:

“First, the sample included only adolescent girls, which limits the generalizability of this study's results to the broader Palestinian adolescent refugee population. Acknowledging the possible nuanced differences between boys and girls in their reaction to the stressors necessitates having a more rigorous validation process and development of items that account for gender-specific experiences. Therefore, the applicability of this scale among boys cannot be assumed and still needs to be established. However, future research to test this scale's validity among males, a mixed sample, and among younger adolescents is necessary.”

3. Third, the rationale for item removal could be strengthened. Several items central to the original SDQ framework (e.g., Obedient, Friend, Fights, Reflective) were excluded due to low factor loadings. While statistically justified, additional theoretical and cultural explanations would help readers understand why these items performed poorly within refugee camp contexts.

We removed items based on the statistical results obtained from the exploratory factor analysis (EFA). EFA was preferred over principal component analysis (PCA) because of their different objectives. EFA assumes the presence of underlying latent factors that explain the relationships among observed variables, whereas PCA is primarily a data-reduction technique. This supports eliminating the items.

After removing poorly performing items, we observed that some remaining items loaded onto factors different from those proposed in the original SDQ structure. We therefore examined whether the newly emerging factor structure and the associated item loadings could be theoretically justified. This evaluation was discussed in the subsection entitled ‘Variations in items’ where we interpreted and explained the observed deviations from the original SDQ factor structure. However, we added additional explanation of the remaining items, attempting to justify their loadings in the final model.

We also added some explanation of the possible reasons why some items (e.g., Loner, Friend, Adults, and Obedient) did not perform well, so they were eliminated. We added this paragraph page 24

“Several items central to the original SDQ framework were excluded through the factor analyses. While statistically justified, an additional explanation of the possible reasons for the poor performance of these items might be needed and requires an understanding of the social relations in Palestinian and Arab culture. Palestinians share a collective memory shaped by prolonged suffering, which strengthens their social ties. Palestinian society, as part of the larger Arab community, is collectivist and shows strong ties to religious elements and the extended family [73]. Collectivist countries prefer closely bonded social (often familial) networks and interpersonal harmony [74]. Therefore, adolescents in this study might not feel isolated or lonely even if they do not have a best friend, due to the potential social relations and networks they have with family, cousins, and neighbors. Instead, weak familial relationships might be the main driver of loneliness [74]. This would explain why items like ‘Friend’ and ‘Loner’ did not perform well in the analyses. It can also apply to ‘Adults’ and ‘obedient’ items. Being obedient and respectful toward adults and the elderly, and socializing time with them, is part of the culture and religious values that children and adolescents are raised on [75], rather than an indication of peer problems. Further research is required to conduct a meticulous exploration of each item's meaning, message, and relevance through the eyes of Palestinian adolescents themselves, leading to a context-specific SDQ version.”

We also added the following at the end of the ‘Variations in items’ subsection Page26:

“Finally, while providing cultural explanation, it is important to avoid speculations in explaining the possible reasons that led to some items being eliminated after the EFA or loading differently compared to the original SDQ. Therefore, further research is required to conduct a meticulous exploration of each item's meaning, message, and relevance through the eyes of Palestinian adolescents themselves, leading to a context-specific SDQ version.”

4. Fourth, convergent validity findings are weak. The reported correlation between SDQ difficulties and WHO-5 well-being was only r = –0.245, and AVE values remained below recommended thresholds. This raises concerns regarding the construct validity of the proposed three-factor model. The discussion should elaborate more critically on the implications of these findings and whether the alternative model is suitable for practical screening purposes.

In the discussion, we highlighted that the three-factor models are not necessarily always better or worse than the original five-factor model (Please refer to the ‘Suggested alternatives’ subsection in the discussion) to support the part in the ‘Conclusion’ section where we stated that utilizing this scale should be done with caution, especially in clinical settings. However, we updated the conclusion statement for more clarity (page 29):

“While the suggested 3-factor alternative provides a more fitting model for assessing difficulties, specifically emotional ones, among adolescent refugees, utilizing this scale should be done with caution, especially in clinical settings, whether for diagnosis or screening.”

Furthermore, we added the following to the discussion in the ‘Convergent and discriminant validity’ subsection (page 27):

“……. This can eventually undermine the construct validity of the proposed three-factor model.

In conclusion, our results imply that utilizing this scale in clinical settings should be done with caution despite its better structure compared to the original SDQ. Although the SDQ is being used as a screening tool in clinical settings to assess adolescents’ mental health and provide information for health care professionals to diagnose psychopathology or disorders [4], there are calls for practitioners to cease using the current form SDQ as a screening tool [4]. A study targeted Aboriginal people in Australia recommended avoiding using the summation of subscale scores for clinical screening [5]. One study in England called for caution in using SDQ to screen for ADHD among children [6]. Therefore, exploring SDQ's psychometric properties in both clinical and community settings is unavoidable to examine its suitability for clinical diagnosis or its usefulness as a screening instrument [7].”

We also added the following in under the reliability subsection in the discussion (page 28):

“Improving the reliability of the scale would potentially improve the potential for use of the scale, including in clinical settings for screening and/or diagnosis. Low reliability coefficients might lead to misclassify true positive cases. Therefore, clinical decisions based on scores calculated from non-reliable scales, especially when dealing with adolescents, should be strongly discouraged [3].”

5. Additionally, the use of self-reported mental health diagnosis and self-perceived difficulties as criterion measures may introduce reporting bias. Inclusion of clinician-administered diagnostic assessments would have provided a stronger external validation criterion.

We fully agree that clinician-administered diagnostic assessments would be the ideal measure for evaluating criterion validity. However, such information was not available and could not be accessed through medical records, even when such records existed. This study was based on a household survey, and data were collected through one-to-one interviews. Consequently, we were only able to obtain self-reported information from participants. We have already addressed this limitation in the strengths and limitations section (page 29):

“Finally, the variables used to evaluate convergent, discriminant, and criterion validity may not be perfect measures to use. For example, the self-reported diagnosis of a psychological or mental illness question might give an underestimation of mental illness levels because many people tend not to seek professional mental health treatment or not to report their actual condition, and this would have an impact on the results."

6. Finally, the manuscript would benefit from language editing. There are several grammatical inconsistencies, repetition, and minor typographical errors throughout the introduction and discussion sections. Careful proofreading would improve readability and overall presentation.

We carefully proofread the manuscript, corrected grammatical and typographical errors, reduced repetition, and improved the overall clarity and readability of the text.

Recommendation: Minor Revision

The study addresses an important gap and offers valuable evidence regarding SDQ use among Palestinian refugee adolescents. However, concerns regarding validity interpretation, theoretical justification of item reduction, generalizability, and presentation should be addressed before the manuscript is suitable for publication.

Reviewer #2:

1. Importance and Justification: There should be a stronger justification for concentrating only on teenage girls. The consequences of leaving out males and the ensuing restrictions on generalizability should be covered by the investigators.

Thank you for this important comment. This study is part of a larger mixed-methods project examining the health and well-being of adolescent girls living in Palestinian refugee camps in the West Bank and Jordan. One of the project's objectives was to assess the mental well-being of adolescent girls using the Strengths and Difficulties Questionnaire (SDQ). Therefore, the study population was defined by the objectives of the parent project rather than the psychometric validation itself. We have clarified this rationale at the beginning of the Methods section. In addition, the issue of generalizability has been addressed in the "Strengths and Limitations" section (please also refer to our response to Reviewer 1, Comment 2).

“This study is part of a larger project addressing the health needs of adolescent girls in Palestine refugee camps in the West Bank and Jordan. The broader project aimed to explore multiple dimensions of adolescent girls' health, including sexual and reproductive health, mental well-being, and the social and environmental factors influencing their health. As part of this project, the Strengths and Difficulties Questionnaire (SDQ) was administered to assess emotional and behavioral well-being and to evaluate its psychometric properties in this understudied population.was conducted in three phases: a qualitative phase to explore these SRH needs, a quantitative phase to measure their extent, and an intervention phase to address them [36,37]. The current study, which validates the SDQ, is based on data collected during the quantitative phase of the project. Given the scope of the research, male adolescents were not included in this study. .”

2. Clarity of Methodology: The research design, sampling methods, participant recruitment processes, and inclusion/exclusion criteria require further clarification.

Thank you for this comment. We revised the Methods section to clarify the cross-sectional study design, stratified sampling across the 29 refugee camps, household recruitment procedures, and the inclusion and exclusion criteria.

3. A more thorough explanation of the item elimination procedure in the alternative models should be provided, together with theoretical support and statistical standards

We modified the Methods section according to your suggestion. Specifically, we provided a more detailed description of the statistical procedure used for iterative item elimination and factor retention. We also clarified that the final proposed factor structure was subsequently evaluated in light of its theoretical and cultural relevance to the context of adolescent Palestinian refugees as follows (page 11):

“We conducted the EFA and CFA iteratively, each time eliminating items that failed to meet the loading threshold (<0.40) in either the EFA or CFA. Subsequent re-evaluation of the factorial structure was conducted. After each round of item elimination, parallel analysis, scree plot inspection, EFA, and CFA were conducted to confirm the optimal number of factors and the stability of the factorial structure [49]. This process was repeated until a psychometrically sound model was obtained. The final proposed model was critically evaluated in terms of its theoretical and cultural foundations to evaluate whether the proposed alternative, comprising the retained items and any variations in their factor loadings, can be theoretically justified and properly interpreted within cultural values, norms, and lived experiences of adolescent Palestinian refugee girls .”

4. Evaluation of Validity: Further discussion is necessary given the WHO-5 Well-Being Index's limited convergent validity. The authors should investigate potential methodological, cultural, or contextual factors underlying this result. Interpretation would be improved by more discussion of the reported AUC values' practical significance and criterion validity.

Thank you for this valuable comment. We have revised the Discussion to provide additional interpretation of the limited convergent validity observed with the WHO-5 Well-Being Index. Specifically, we discuss how the SDQ and WHO-5 assess related but distinct constructs (emotional and behavioral difficulties versus positive subjective well-being) and how differences in their recall periods may have contributed to the weak association. We also expanded the interpretation of the criterion validity findings by discussing the practical significance of the AUC values, sensitivity, and specificity. In particular, we clarify that the poor AUC values for the WHO-5 outcomes and the moderate sensitivity and specificity indicate limited discrimination and limited criterion validity when the WHO-5 is used as the external criterion.

5. Issues with Reliability: The three-factor model showed better fit indices, but its dependability was only moderate. The authors should discuss the suitability of the suggested model for clinical or screening applications.

We would kindly ask you to refer to our response to Comment 4 from Reviewer 1, where we addressed the suitability of the SDQ for clinical or screening applications.

6. Minor suggestions

1. Consider creating a summary table with the final three-factor model's 15 items that were kept.

We appreciate the reviewer’s suggestion. However, as the items are already presented in Table 6, we feel that reproducing them in an additional table would be redundant and may not provide substantial additional information.

2. The study's application might be increased with a brief explanation of the practical implications for mental health screening programs in refugee situations.

Thank you for this valuable suggestion. We have expanded the Conclusion section to better highlight the practical implications of our findings for mental health screening in refugee settings. Specifically, we emphasize the importance of using psychometrically robust and contextually relevant screening tools to support accurate identification of adolescents who may require further mental health assessment.

We added this to the conclusion (page 29)

“While the suggested 3-factor alternative provides a more fitting model for assessing difficulties, specifically emotional ones, among adolescent refugees, utilizing this scale should be done with caution, especially in clinical settings, whether for diagnosis or screening. Our findings highlight the importance of validating mental health screening instruments before their implementation in humanitarian settings. These findings underscore the need for psychometrically robust and contextually relevant screening tools to support accurate identification of adolescents who may benefit from further mental health assessment and appropriate referral”

Reviewer #3:

1. It's a good attempt to highlight the SDQ model with its pros and cons. This study has used data from a wider study on the targeted population. It needs to present the basic socio-economic profile of the respondents, i.e., adolescents and their families, for better understanding by readers.

Thank you for this comment. We have revised Table 2 to provide a clearer socioeconomic profile of the adolescent girls and their families. The additional variables include the nature of the camp community, family type, and household standard of living.

2. Reviews have been done appropriately to supplement a sufficient argument to the core argument. However, study objectives and core research questions have not been mentioned properly.

We thank the reviewer for this comment. To clarify the study objectives and research questions, we have revised the Introduction to explicitly state that the study aimed not only to validate the SDQ among Palestinian refugee adolescents but also to evaluate whether the original SDQ factor structure is appropriate for this population and to explore alternative models that may provide a better fit for the cultural context (page 7).

3. In Methodology, it is written that the study has been conducted in three phases – Qualitative, Quantitative, and Intervention Phases; further, it mentioned the "Structures Survey Tools" that were used to collect data. It's better to mention "semi-structured tools", or it may be justified by the author.

This study only uses data from the quantitative phase. So, the questions used in the survey tool are structured. In-depth interviews were used for the qualitative phase, which is not in the scope of this manuscript. You can refer to the qualitative paper published from the qualitative phase using this link: https://doi.org/10.1080/26410397.2022.2111793

4. The analysis part is well explained, giving priority to validating each point of the SDQ, whether they are worth probing or not, thus drawing the acceptance or modification of the model (ADQ) itself. At the same time, can it be more reader-friendly? Extensive analysis creates more critical understanding for the readers.

Thank you. We have revised the language throughout the Results section to improve readability and flow while maintaining the methodological detail necessary to support the psychometric analyses. We also simplified several descriptions to make the analytical process easier to follow for readers.

5. Limitations have been well placed. The SDQ model has its own limitations, and could be modified or replaced by a better model over time and tested in different settings.

We agree with this notion. As noted in the manuscript, we recommend further refinement and adaptation of the SDQ to better fit the context of Palestinian refugees. The SDQ may be a context-sensitive measure, and items that perform well in Western populations may not necessarily demonstrate the same validity in other cultural contexts.

Attachment Submitted filename: Response to Reviewers 1.2.docx

# Peer review: Decision Letter 1

**DOI:** 10.1371/journal.pone.0349999.r003

**Authors:** Harikrishnan U

1 Sep 2026

Exploring psychometric properties of the Strengths and Difficulties (SDQ) among Adolescent Palestinian refugee girls in Palestine and Jordan

PONE-D-26-18251R1

Dear Dr. Ghandour,

We’re pleased to inform you that your manuscript has been judged scientifically suitable for publication and will be formally accepted for publication once it meets all outstanding technical requirements.

Within one week, you’ll receive an e-mail detailing the required amendments. When these have been addressed, you’ll receive a formal acceptance letter and your manuscript will be scheduled for publication.

An invoice will be generated when your article is formally accepted. Please note, if your institution has a publishing partnership with PLOS and your article meets the relevant criteria, all or part of your publication costs will be covered. Please make sure your user information is up-to-date by logging into Editorial Manager at Editorial Manager® and clicking the ‘Update My Information' link at the top of the page. For questions related to billing, please contact billing support.

If your institution or institutions have a press office, please notify them about your upcoming paper to help maximize its impact. If they’ll be preparing press materials, please inform our press team as soon as possible -- no later than 48 hours after receiving the formal acceptance. Your manuscript will remain under strict press embargo until 2 pm Eastern Time on the date of publication. For more information, please contact onepress@plos.org.

Kind regards,

Harikrishnan U, Ph.D

Academic Editor

PLOS One

Additional Editor Comments (optional):

Reviewers' comments:

Reviewer's Responses to Questions

**Comments to the Author**

Reviewer #3: All comments have been addressed

Reviewer #4: All comments have been addressed

**********

2. Is the manuscript technically sound, and do the data support the conclusions?

Reviewer #3: Yes

Reviewer #4: Yes

**********

3. Has the statistical analysis been performed appropriately and rigorously?

Reviewer #3: Yes

Reviewer #4: Yes

**********

4. Have the authors made all data underlying the findings in their manuscript fully available?

The PLOS Data policy

Reviewer #3: Yes

Reviewer #4: Yes

**********

5. Is the manuscript presented in an intelligible fashion and written in standard English?

Reviewer #3: Yes

Reviewer #4: Yes

**********

Reviewer #3: The author responded to all our comments. Thus, we recommended the acceptance/further procedure of the article.

Reviewer #4: The article is relevant to the review topic and provides useful information. The objectives and methodology are clearly presented. Overall, the article appears suitable for further screening and full-text review.

**********

what does this mean?). If published, this will include your full peer review and any attached files.

If you choose “no”, your identity will remain anonymous but your review may still be made public.

**Do you want your identity to be public for this peer review?**  For information about this choice, including consent withdrawal, please see our Privacy Policy

Reviewer #3: **Yes:**  Golak B Patra

Reviewer #4: No

**********

# Editor report: Acceptance letter

**DOI:** 10.1371/journal.pone.0349999.r004

**Authors:** Harikrishnan U

PONE-D-26-18251R1

PLOS One

Dear Dr. Ghandour,

I'm pleased to inform you that your manuscript has been deemed suitable for publication in PLOS One. Congratulations! Your manuscript is now being handed over to our production team.

At this stage, our production department will prepare your paper for publication. This includes ensuring the following:

* All references, tables, and figures are properly cited

* All relevant supporting information is included in the manuscript submission,

* There are no issues that prevent the paper from being properly typeset

You will receive further instructions from the production team, including instructions on how to review your proof when it is ready. Please keep in mind that we are working through a large volume of accepted articles, so please give us a few days to review your paper and let you know the next and final steps.

Lastly, if your institution or institutions have a press office, please let them know about your upcoming paper now to help maximize its impact. If they'll be preparing press materials, please inform our press team within the next 48 hours. Your manuscript will remain under strict press embargo until 2 pm Eastern Time on the date of publication. For more information, please contact onepress@plos.org.

You will receive an invoice from PLOS for your publication fee after your manuscript has reached the completed accept phase. If you receive an email requesting payment before acceptance or for any other service, this may be a phishing scheme. Learn how to identify phishing emails and protect your accounts at https://explore.plos.org/phishing.

If we can help with anything else, please email us at customercare@plos.org.

Thank you for submitting your work to PLOS One and supporting open access.

Kind regards,

PLOS One Editorial Office Staff

on behalf of

Dr. Harikrishnan U

Academic Editor

PLOS One
